# Evidences of topological nodal line semimetal in Mn_3_GaC: Anomalous Hall effect, thermal transport and DFT studies

**DOI:** 10.1038/s41598-025-10563-4

**Published:** 2025-09-29

**Authors:** Sunil Gangwar, Amarjyoti Choudhury, Tulika Maitra, C. S. Yadav

**Affiliations:** 1 School of Physical Sciences, Indian Institute of Technology Mandi, Kamand, 175075 Mandi, H.P. India; 2 Department of Physics, Indian Institute of Technology Roorkee, 247667 Roorkee, Uttrakhand India; 3 Center for Quantum Science and Technologies, Indian Institute of Technology Mandi, Kamand, 175075 Mandi, H.P. India

**Keywords:** Topological nodal line semimetal, Anomalous Hall effect and thermal transport, Antiperovskites, Physics, Condensed-matter physics

## Abstract

The non-trivial electronic transport in magnetic topological materials have attracted significant attention. Here, we present evidences of a topological nodal line semimetal in antiperovskite $\hbox {Mn}_{{3}}$GaC along with the experimental studies such as the anomalous Hall effect (AHE), Kondo effect and thermal transport properties (Seebeck and Nernst effects). The upturn in the low-temperature electrical resistivity follows Hamann expression with the Kondo temperature $T_{K}$ = 16 K. The scaling analysis of the anomalous Hall conductivity ($\sigma _{AHE}$) suggests that the AHE in $\hbox {Mn}_{{3}}$GaC is primarily governed by coexistence of both intrinsic Berry curvature and skew scattering mechanisms. The experimentally observed value of $\sigma _{AHE}$ ($\sim$ 50 $\Omega ^{-1} \text {cm}^{-1}$) is close to the theoretically calculated value. The low temperature Seebeck data suggests the presence of significant contribution of electron–magnon scattering, and a large value of Nernst coefficient is consistent with finite Berry curvature effects in $\hbox {Mn}_{{3}}$GaC. The electronic band structure calculations with spin-orbit coupling, shows the formation of a drumhead-shaped surface states, and the existence of finite number of Weyl nodes, in consistence with the experimental findings.

## Introduction

The study of non-trivial properties in topological semimetals is a topic of great interest in both theoretical and experimental condensed matter physics. Topological semimetals are categorised into Dirac semimetals (DSMs), Weyl semimetals (WSMs) and nodal line semimetals (NLSs); on the basis of the dimension of the band touching in momentum space^1,2^. The DSMs and WSMs have zero-dimension (0D) band crossing near the Fermi level (FL), whereas in NLSs valance and conduction bands cross to form a 1D loops or lines in the momentum space^1–3^. The time reversal symmetry (TRS) and inversion symmetry (IS) are preserved in DSMs and they exhibit fourfold degenerate Dirac points^4^. In WSMs, degeneracy of bands splits by breaking either TRS or IS, resulting in band-crossing points known as Weyl points with opposite chirality^5,6^. Additional symmetries, such as mirror reflection, are required in NLSs to ensure the topological protection of line-like contacts between the conduction and valence bands, beyond mere translational symmetry^6^. These bands crossing exert a significant impact on the electronic properties of topological semimetals, and exhibit intriguing properties such as drumhead-like surface states^1,2^, anomalous Hall effect (AHE)^7^ and high-temperature superconductivity^6^. Although Dirac and Weyl semimetals have been thoroughly investigated, exploration of nodal-line semimetals has been limited due to the absence of a suitable material platform. Few materials such as ZrSiTe^1^, CaTX (T = Ag, Cd; X = As, Ge)^2^, CaCdSn^3^ and $\hbox {PbTaSe}_{{2}}$^6^ are predicted to show nodal line topological properties. In recent years, antiperovskites compounds such as $\hbox {Ca}_{{3}}$PbO^8^, $\hbox {Sr}_{{3}}$PbO^9^, $\hbox {Sr}_{{3}}$SnO^10^ and $\hbox {Cu}_{{3}}$PdN^11^ have gained research interest due to the prediction of their topological surface states. Among these, Mn-based antiperovskite have attracted special interest due to their complex magnetic ordering and non-trivial properties like AHE and anomalous Nernst effect^12^. In particular, the AHE is one of the intriguing transport property in magnetic materials, in which the transverse voltage arises from the longitudinal current in the presence of spin orbit coupling (SOC)^7^. Moreover, nodal lines can act as important sources of Berry curvature, which leads to the AHE. The Berry curvature acts as a fictitious magnetic field in momentum space, influencing intrinsic anomalous Hall conductivity as well as a large Nernst coefficient in the syatem.

Teicher *et al.* showed that $\hbox {Mn}_{{3}}$ZnC belongs to the family of NLSs with nodal loops, isolated Weyl points and drum-head surface states^13^. $\hbox {Mn}_{{3}}$ZnC shows paramagnetic (PM) to ferromagnetic (FM) transition at $T_{C}$ $\sim$ 380 K, followed by an antiferromagnetic (AFM) transition at $T_{N}$ $\sim$ 233 K^13^. In $\hbox {Mn}_{{3}}$SnC, both FM and AFM states are reported to coexist below $T_{C}$ $\sim$ 280 K^14^. Similar to these, $\hbox {Mn}_{{3}}$GaC, shows PM to FM transition at $T_{C}$ $\sim$ 248 K, followed by a FM to AFM transition at $T_{N}$ $\sim$ 164 K^15,16^. In this family of compounds, the magnetic state is governed by the presence of distorted $\hbox {Mn}_{{6}}$C octahedra with long and short Mn-Mn bonds. X-ray absorption fine structure study suggested that Mn-Mn bond length varies, while Mn-C distance shows negligible variation with distortion in $\hbox {Mn}_{{6}}$C octahedra^16,17^. Owing to its magnetic ground states and antiperovskite structure, $\hbox {Mn}_{{3}}$GaC is expected to show non-trivial transport properties accompanied by the Weyl nodes.

Here, we report comprehensive magneto-transport, thermoelectric studies and electronic band structure studies on $\hbox {Mn}_{{3}}$GaC. Interestingly, the low temperature electrical resistivity exhibits Kondo effect. The transverse electrical resistivity shows contributions from both ordinary and anomalous Hall effects. The anomalous Hall conductivity ($\sigma _{AHE}$) shows sharp anomalies at the magnetic transition, and AHE is found to have contributions from both intrinsic finite Berry curvature and extrinsic skew scatterings. Furthermore, The low temperature Seebeck data suggests the magnon drag contribution caused via a both FM and AFM spin magnon scatterings. We also discuss the thermoelectric properties through temperature dependent Seebeck and Nernst coefficient data in zero temperature limit. The first principle calculations shows band touching points, giving rise to drumhead like surface states, confirming the existence of topological nodal-line phase in $\hbox {Mn}_{{3}}$GaC.

## Results and discussion

### Experimental results

**Fig. 1:**
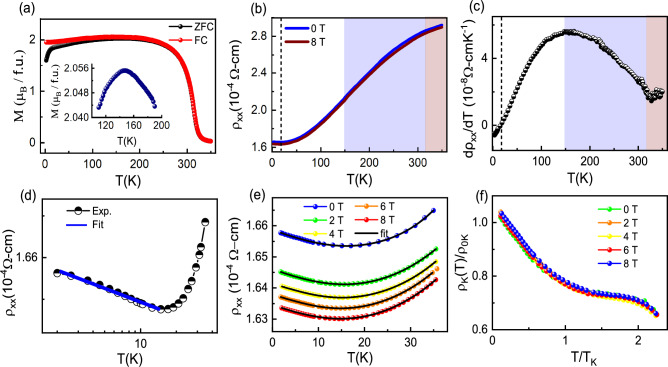
(**a**) M(T) measured under ZFC and FC protocols at *B* = 0.1 T. The inset shows a zoomed region around 150 K. (**b**) $\rho _{xx}$ vs *T* at B = 0, 8 T. (**c**) First order derivative of $\rho _{xx} (T)$. (**d**) Low temperature $\rho _{xx}$ in logarithmic *T* scale. The solid blue line represents the linear fit at low temperature. (**e**) $\rho _{xx} (T)$ below 35 K at several magnetic field. Black line shows the Kondo fit. (**f**) Universal Kondo behavior of normalized resistivity vs *T* scaled to the Kondo temperature $\hbox {T}_{{K}}$ taken at different magnetic fields. Black dashed line indicates the Kondo region, blue and red shaded colour region indicate the ferromagnetic and paramagnetic state respectively.

Figure 1(a) shows temperature dependent magnetization (M) of $\hbox {Mn}_3$GaC measured at 0.1 T field during zero- field cooling (ZFC) and field cooled warming (FCW) modes. It shows a FM transition at $T_{C} \sim$ 310 K. Inset of the Figure 1(a) shows the zoomed region around $T_{N}$ $\sim$ 150 K, which shows a reduction in magnetization indicative of an AFM transition. Neutron diffraction studies on $\hbox {Mn}_3$SnC and $\hbox {Mn}_3$ZnC also suggest the setting of the non-collinear AFM ordering of Mn atoms at low temperature^18,19^. The variation in carbon concentration can expand or reduce the volume of the unit cell, resulting in Mn-Mn bond length changes in $\hbox {Mn}_{{3}}$GaC. It is to mention here that the stoichiometry of carbon affect the size of unit cell and the hybridization of the Mn orbitals bands leading to a reduced AFM transition in the carbon deficient compound $\hbox {Mn}_{{3}}$GaC^16,20^.

Figure 1(b) shows the temperature dependence of the longitudinal resistivity ($\rho _{xx}(T)$) in the zero and 8 T field. The $\rho _{xx}(T)$ shows metallic behavior with the value of the residual resistivity ratio (RRR), which is defined as $\rho _{(T = 300K)}/\rho _{(T = 1.8 K)}$ is $\sim$ 1.8. This RRR value is comparatively on the lower side for a metallic system and shows the significant role of internal defects and grain boundaries on the conduction of the charge carriers. However, this value is similar to the RRR values reported in this family of compounds such as RRR $\sim$ 2.8 ($\hbox {Mn}_{{3}}$GaC)^16^, RRR $\sim$ 1.2 ($\hbox {Mn}_{{3}}$SnC)^18^ and RRR $\sim$ 3.5 ($\hbox {Mn}_{{3}}$ZnC)^21^. It should be mentioned that high-quality carbide materials (high RRR value) are difficult to prepare. The first-order derivative of resistivity shows a change in slope around $\sim$ 150 K and $\sim$ 310 K, coinciding with the magnetic transitions, as shown in Figure 1(c). The $\rho _{xx}(T)$ exhibits an upturn below $\sim$ 20 K. This low temperature upturn can be explained by the electron-electron interaction (EEI), weak localization (WL) and the Kondo effect mechanisms^22^. For the studied materials, $\rho _{xx}$-*T* follows $T^{n/2}$ (n = 3/2, 2, 3 depending on the scattering mechanism) relation in the WL effect^22,23^, a $T^{1/2}$ dependence in EEI^22–24^ and a ln *T* dependence in Kondo effect^22,25–27^. We fitted the experimental $\rho _{xx} (T)$ data using scaling relations of WL and EEI, however, both effects displayed the poor agreement fitting with the experimental data. Figure 1(d) shows the low temperature resistivity in ln *T* scale, where the linear fit follows experimental data, as expected in the Kondo effect. The Kondo effect originates from the spin scattering between the localized magnetic moments and the conduction electrons^25–27^. The Kondo effect in the metal arises due to the interaction of conduction electrons with magnetic impurity. The conduction electrons scatter with either the localised moments or the interstitial sites/defects (introducing magnetic moment). The elemental analysis of the compound suggests the presence of additional interstitial Mn atoms ($\hbox {Mn}_{3.08} \hbox {Ga}_{0.97}\hbox {C}_{0.95}$). The interaction of the conduction electrons with the local magnetic moment introduced by the unpaired d-electrons of interstitial Mn atoms, might be a reason for the Kondo scattering in this magnetically ordered system. The presence of additional interstitial Mn species in this compound might be a reason of observation of the Kondo-like behaviour. The temperature range 1.8–35 K, includes the temperature below and above the observed $\rho _{xx}(T)$ minimum, and can be examined by the Hamann’s expression^22,28^.

1 $$\begin{aligned} \rho (T)= & \rho _0 + aT^2 + bT^5 +\rho _k(T) \end{aligned}$$

2 $$\begin{aligned} \rho _k(T)= & \rho _{0K}\left[ 1-ln\left( \frac{T}{T_K}\right) \left\{ {ln}^2\left( \frac{T}{T_K}\right) +s\left( s+1\right) ^2\right\} ^{-0.5}\right] \end{aligned}$$

**Fig. 2:**
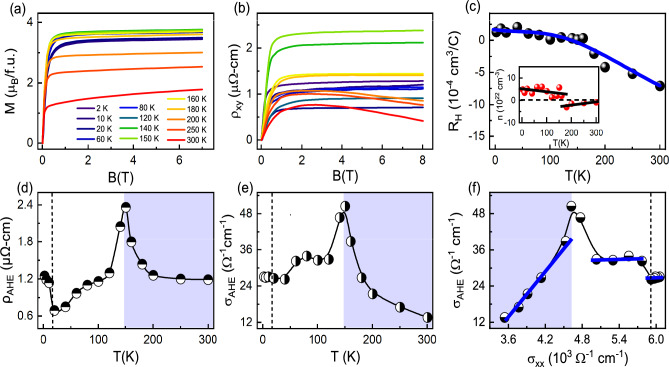
Magnetic field dependent magnetization M vs B at different temperatures. (**b**) Magnetic field dependent Hall resistivity$\rho_{xy}$ at different temperatures as same in magnetization. (**c**) Temperature dependent normal Hall coefficient$R_{H}$. The inset shows the carrier density*n* as a function of temperature. (**d**) Temperature dependent anomalous Hall resistivity$\rho _{AHE}$. (**e**) Temperature dependent anomalous Hall conductivity$\sigma _{AHE}$. (**f**) Plot between$\sigma _{AHE}$ and longitudinal conductivity$\sigma _{xx}$, and the fitting is shown by solid blue line. Black dashed line and blue shaded colour region indicate the Kondo region and ferromagnetic state respectively.

where $\rho _{0}$ is the residual resistivity. The $\sim$ $T^{2}$ term corresponds to electron-electron scattering or electron-magnon scattering with the FM spin waves^29^. The $\sim$ $T^{5}$, *s*, $T_{K}$ and $\rho _{0K}$ represent the electron-phonon scattering contribution, spin of magnetic impurity, Kondo temperature and temperature independent Kondo resistivity respectively. Figure 1(e) shows the experimental data at different magnetic fields, fitted with the expression (1). The parameters obtained from the fitting are $T_{K}$ = 16 ± 2.6 K, average impurity spin per Mn is 0.19 ± 0.016, $\rho _{0}$ = $(1.6 \pm 0.0026)\times {10^{-4}}$ $\Omega$-cm, *a* = $(9.03 \pm 0.14)\times {10^{-8}}$ $\Omega$-$\hbox {cmK}^{-2}$, *b* = $(1.24 \pm 0.12)\times {10^{-12}}$ $\Omega$-$\hbox {cmK}^{-5}$ and $\rho _{0K}$ = $(7.97 \pm 0.14)\times {10^{-5}}$ $\Omega$-cm. The low value of spin indicates that the only a small amount of localized magnetic moments of Mn ions induce the Kondo effect^26,28^. The $\rho _{xx} (T)$ curves at different magnetic fields can be scaled with the universal Kondo behaviour by using the fitting parameters. Figure 1(f) shows the normalized Kondo resistivity ($\rho _{K}$(*T*))/$\rho _{0K}$ vs *T*/$T_{K}$ at different magnetic fields. It is found that all the experimental curves overlaps with each other, confirming the validity of the Kondo behavior^25,27^. We also plot longitudinal resistivity as a function of applied magnetic field at different temperatures (See supplementary Figure S4).

The Hall resistivity was measured in the temperature range of 2-300 K up to an 8 T magnetic field. Generally, the total Hall resistivity of a magnetic system consists contributions from ordinary Hall and anomalous Hall, and can be expressed as

3 $$\begin{aligned} \rho _{xy}(B) = \rho _{0} + \rho _{AHE} = R_{H}B + R_{S}M_{S} \end{aligned}$$

where $\rho _{0}$, $\rho _{AHE}$, $R_{H}$, $R_{S}$, *B* and $M_{S}$ represent the ordinary Hall resistivity, anomalous Hall resistivity, ordinary Hall coefficient, anomalous Hall coefficient, applied magnetic field and saturated magnetization respectively^30–35^. AHE is understood to arise in the materials due to intrinsic and extrinsic mechanisms. Whereas the asymmetric scattering of the conduction electrons in the presence of spin orbit coupling (SOC) or impurities give extrinsic contribution to AHE, the intrinsic mechanism is related to the Berry curvature associated with the occupied electronic Bloch electrons^30–34^. The skew scattering and side jump contributions are involved in the extrinsic mechanism. Skew scattering can be caused by various factors, including defects, impurities, electron-phonon interactions, spin-dependent scattering, and the presence of spin clusters^36^. It is challenging to experimentally separate the contributions from the side jump and intrinsic Berry curvature, as both exhibit the same scaling behavior. For a low RRR value material, AHE may have significant contribution from the skew scattering, and it may pose difficulty in extracting the intrinsic contribution from the Berry curvature. The linear scaling relation between $\sigma _{AHE}$ and the longitudinal electrical conductivity $\sigma _{xx}$ ($\sigma _{AHE}$ $\sim$ $\sigma _{xx}$) indicates the skew scattering contribution. In intrinsic mechanism, $\sigma _{AHE}$ remains independent of $\sigma _{xx}$ and temperature^32–34^. We first anti-symmetrized the raw $\rho _{xy}(H)$ data to remove the longitudinal component, which may arise due to the misalignment of contacts^37^. The field dependent $\rho _{xy}$ is plotted in 2 (b) at different temperatures up to a magnetic field of 8 T. The $\rho _{xy}(H)$ rises sharply with magnetic field up to 1 T, which can be attributed due to the AHE. However, at higher fields (> 1 T), linear curves are observed due to the ordinary Hall effect^30,31^. The $\rho _{xy}(H)$ curves exhibit a similar field dependency to that observed in *M*(*H*) (see Figure 2(a)), confirming the presence of AHE in the $\hbox {Mn}_{{3}}$GaC. Figure 2(c) shows the temperature variation of $R_{H}$, calculated from the slope of the high field $\rho _{xy}(H)$ curve^30,31^. The negative and positive signs of $R_{H}$ indicate both electron and hole types of charge carriers in the transport. The inset of Figure 2(c) shows the carrier concentration *n* as a function of temperature, calculated from the slope of the high magnetic field data using single band model with expression $R_{H}$ = 1/$\textit{ne}$. The value of *n* is found to be $\sim$ 2.3$\times 10^{22}$ $\hbox {cm}^{-3}$ and $\sim$ 0.81$\times 10^{22}$ $\hbox {cm}^{-3}$ at 2 and 300 K, respectively. The $\rho _{AHE}$ is calculated from the extrapolation of high field $\rho _{xy} (H)$ curves to zero field on the y-axis^30,31^. Temperature dependent $\rho _{AHE}$ is plotted in Figure 2(d). On approaching towards $T_{N}$, the magnetic transition significantly alter the overall characteristics of the $\rho _{AHE} (T)$. The $\rho _{AHE} (T)$ initially rises as the temperature increases, reaching its peak value of 2.36 $\mu \Omega$-cm at $\sim$ 150 K, then sharply decreases as the temperature continues to rise, reaching a low value of $\sim$ 0.65 $\mu \Omega$-cm around 20 K. The detection of an increase at low temperature in $\rho _{AHE}(T)$ correlates with Kondo effect, as suggested in literature^34^. In order to investigate the origin of AHE in $\hbox {Mn}_{{3}}$GaC, we examine the scaling relation between $\sigma _{AHE}$ and $\sigma _{xx}$. The magnitude of $\sigma _{AHE}$ can be calculated using the relation as^32–35^:

4 $$\begin{aligned} \sigma _{AHE} = \frac{\rho _{AHE}}{\rho _{AHE}^2 + \rho _{xx}^2} \end{aligned}$$

**Fig. 3:**
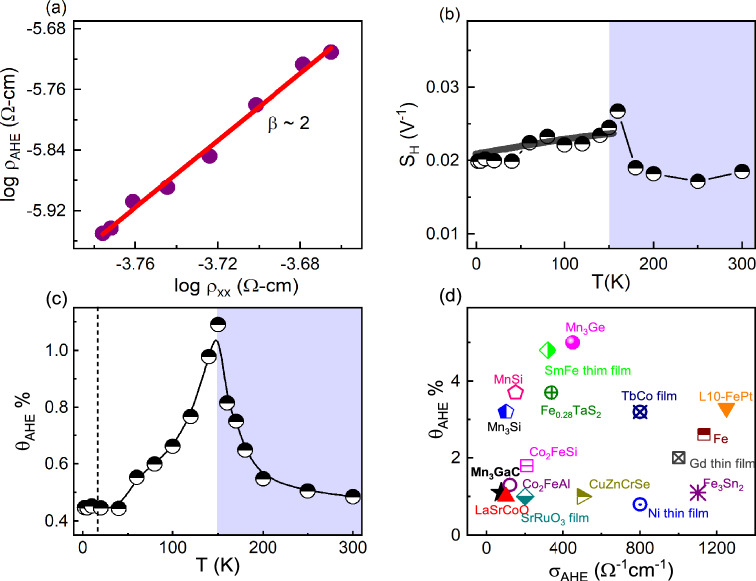
(**a**) Temperature dependent anomalous Hall angle $\theta _{AHE}$. (**b**) Comparison of $\theta _{AHE}$ between $\hbox {Mn}_{{3}}$GaC other AHE materials. The references used to compile the presented data are given in the Supplementary Information Table S3.

Figure 2(e) shows the variation of $\sigma _{AHE}$ with temperature. Here, we observed a relatively low value of $\sigma _{AHE}$, although it is comparable to studied magnetic material $\hbox {Co}_{{2}}$TiGe^38^. The value of AHC depends on several factors, such as the strength of SOC, magnetization, band structure, Berry curvature, etc. The low magnetization or weak ferromagnetism observed in $\hbox {Mn}_{{3}}$GaC may be responsible for the low AHC. Similarly, weak SOC show a small anomalous Hall response because the intrinsic Berry curvature contribution is weak. The maximum value of $\sigma _{AHE}$ is $\sim$ 50 $\Omega ^{-1} \text {cm}^{-1}$ at 150 K, which reduces to $\sim$ 13 $\Omega ^{-1} \text {cm}^{-1}$ at 300 K. To separate out the extrinsic and intrinsic contributions, we plot $\sigma _{AHE}$ as a function of $\sigma _{xx}$ in Figure 2(f). In the temperature range of $2 \le T \le 150 K$, $\sigma _{AHE}$ does not depend on $\sigma _{xx}$. Moreover, it is also noted that the $\sigma _{AHE}$ remains approximately 28 $\Omega ^{-1} \text {cm}^{-1}$ at 2 K and exhibits only a slight variation up to 120 K (32 $\Omega ^{-1} \text {cm}^{-1}$) as shown in Figure 2(e). This suggests that the $\sigma _{AHE}$ remains unaffected by changes in temperature, as well as $\sigma _{\text {xx}}$, implying that the AHE is governed by the intrinsic Berry curvature mechanism^32–35^. In literature, it has been seen that Kondo systems form coherent states at low temperature which are responsible for skew scattering. However, in $\hbox {Mn}_{{3}}$GaC, intrinsic contribution dominants over skew scattering in Kondo region, similar observation also has been found in USbTe^34^. On the other hand, in the temperature range of $150 \le T \le 300 K$, $\sigma _{\text {AHE}}$ follow linear dependency with $\sigma _{xx}$, indicates the skew scattering contribution. Thus, a detailed analysis of AHE of the $\hbox {Mn}_{{3}}$GaC indicates that both intrinsic and extrinsic contributions are involved in the origin of AHE. To confirm the Berry curvature contribution, we analyzed the AHE by examining the relationship between $\rho _{AHE}$ and $\rho _{xx}$, according to the scaling relation $\rho _{AHE} \propto \rho ^{\beta }_{xx}$, where $\beta$ is the positive exponent^35^. The value of $\beta$ = 1 and 2 indicates that AHE originate due to the intrinsic Berry curvature or side jump mechanism and skew scattering mechanism, respectively. In Fig. 3(a), we plot $\rho _{AHE}$ and $\rho _{xx}$ on double logarithmic scale in the temperature range of 20–150 K. Below 20 K, the Kondo effect causes an increase in $\rho _{AHE}$; therefore, our analysis is limited to temperatures above 20 K. Above 150 K, the magnetic phase transition significantly influences the system, leading to a rapid decrease in $\rho _{AHE}$ upon temperature increases. Consequently, it becomes challenging to apply the scaling relation between $\rho _{AHE}$ and $\rho _{xx}$. To extract the exponent $\beta$, we fit logarithmic $\rho _{AHE}$ and $\rho _{xx}$ data, and the value is found to be $\sim$ 2 as shown by solid red line, indicating that the AHE is primarily governed by the intrinsic Berry curvature mechanism. Further, we calculate anomalous scaling factor $S_{H}$, related to the relative magnitude of the anomalous Hall current with respect to the magnetization. It is known that for Berry curvature dominant contribution, $S_{H}$ should be constant and temperature independent. In fig. 3(b), we plot $S_{H}$ as a function of temperature, exhibits nearly temperature independent behavior in the temperature range of 20–150 K. Above 150 K, $S_{H}$ exhibits a strong temperature dependence, indicating the evolution of the skew scattering contribution in the system.

Further, we calculated the anomalous Hall angle ($\theta _{AHE} = \sigma _{AHE} / \sigma _{xx}$), and have shown in Figure 3(c) as a function of temperature. $\theta _{AHE}$ measures the relative contribution of anomalous Hall current with respect to the longitudinal current^33^. As the temperature increases, $\theta _{AHE}$ initially rises from 0.45 % at 2 K, peaking at roughly 1.1 % around 150 K, then decreases as the temperature exceeds $T_{N}$. In Figure 3(d), we present $\theta _{AHE}$ and $\sigma _{AHE}$ values for some magnetic materials^39–49^. The value of $\theta _{A}$ for $\hbox {Mn}_{{3}}$GaC are similar to that for $\hbox {Co}_{{2}}$FeAl, $\hbox {Co}_{{2}}$FeSi and 2-3 times smaller than for $\hbox {Mn}_{{3}}$Si and MnSi. The clean band structure of topological materials can produce both large $\sigma _{AHE}$ and $\theta _{A}$ as expected in the literature for $\hbox {Co}_{{3}}\hbox {Sn}_{{2}}\hbox {S}_{{2}}$ compound^33^. Thus, this observation suggests that when AHC is low, $\hbox {Mn}_{{3}}$GaC produce a low value of $\theta _{AHE}$.

**Fig. 4:**
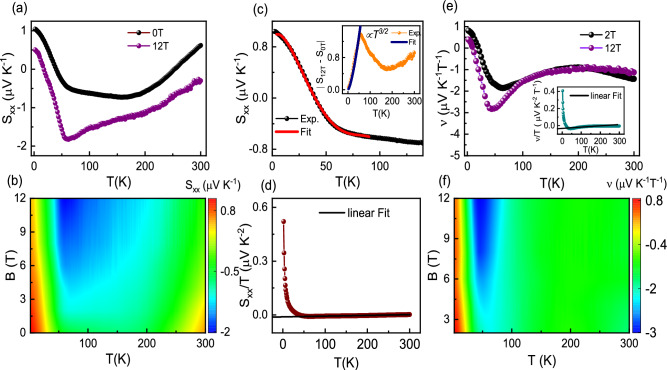
(**a**) Temperature dependent Seebeck coefficient $S_{xx}$ at zero and 12 T field. (**b**) Contour shows $S_{xx}$ as a function of temperature at different magnetic fields (**c**) The low-temperature $S_{xx}$ is fitted up to 90 K, using the expression $S_{xx} (T) = S_{0} + S_{3/2}T^{3/2} + S_{3}T^{3} + S_{4}T^{4}$. The inset shows the temperature dependent $S_{xx}$ measured in presence and absence of the magnetic field of 12 T. (**d**) $\frac{S_{xx}}{T}$ as a function temperature in zero-field. The inset shows the $\frac{\nu }{T}$ as function temperature at 2 T field. (**e**) Temperature dependent Nernst coefficient $\nu$ at 2 T and 12 T field. (**f**) Contour shows $\nu$ as a function of temperature at different magnetic fields.

Figure 4(a) shows Seebeck coefficient ($S_{xx} = \frac{E_{x}}{|\nabla T_{x}|}$) as a function of temperature at 0 and 12 T field. $S_{xx}$(*T*) decreases with decreasing temperature and reaches a peak with a value of −0.5 $\mu VK^{-1}$ around $\sim$ 55 K, which can be attributed to the drag mechanism (phonon drag effect/magnon drag effect)^50–54^. Point defects in the system can act as scattering centers and reduce the mobility of the thermal charge carriers, which may result in the increase in the Seebeck coefficient.The contour plot in Figure 4(b) depicts the effect of magnetic field on $S_{xx}(T)$. It is noted that the peak value around 55 K shows significant enhancement with increasing the magnetic field. Generally, the magnetic field dependence in magnonic Seebeck coefficient is stronger than phononic Seebeck coefficient^54^. And sometime it is difficult to observe a phonon drag contribution in polycrystalline samples due to the presence of large crystal defects^50^. The magnon drag contribution is directly related to the magnon specific heat^50,54^. However, distinguishing the contribution from magnon drag in the presence of AFM-magnons and phonon drag proves challenging, as both exhibit a same temperature dispersion $T^{3}$ in low-temperature regimes. The magnon drag effect due to the FM-magnons can be related with temperature dependence of $T^{3/2}$^50,54^. In magnetic systems, Seebeck coefficient can be originated from the electron diffusion, magnon drag effect or phonon drag effect and spin wave fluctuations.Therefore, we fitted our data using the expression^55,56^

5 $$\begin{aligned} S_{xx} (T) = S_{0} + S_{3/2}T^{3/2} + S_{3}T^{3} + S_{4}T^{4}, \end{aligned}$$

where $S_{0}$ is a constant term, $S_{3/2}T^{3/2}$ represents the FM-magnons contribution, $S_{3}T^{3}$ represents the AFM-magnons contribution and last term represent the spin wave fluctuations as shown in Figure 4(c). The corresponding fitting parameters are $S_{0}$ = $1.10 \pm 0.0078$ $\mu \hbox {VK}^{-1}$, $S_{3/2}$ = $(-5.65 \pm 0.066)\times {10^{-3}}$ $\mu \hbox {VK}^{-2.5}$, $S_{3}$ = $(5.73 \pm 0.11)\times {10^{-6}}$ $\mu$V $K^{-4}$ and $S_{4}$ = $(-1.34 \pm 0.22)\times {10^{-8}}$ $\mu \hbox {VK}^{-5}$. In order to estimate the dominant scattering mechanism at low temperature, we calculated all the terms at *T* = 10 K, which are found to be $S_{0}$ = 1.10 $\mu \hbox {VK}^{-1}$, $S_{3/2}T^{3/2}$ = −0.178 $\mu \hbox {VK}^{-1}$, $S_{3}T^{3}$ = 0.00573 $\mu \hbox {VK}^{-1}$ and $S_{4}T^{4}$ = −0.000134 $\mu \hbox {VK}^{-1}$. Thus, the absolute value of $S_{3/2}T^{3/2}$ is $\sim$ 3 orders of magnitude higher than that of $S_{3}T^{3}$, indicating the dominance of FM-magnon over the AFM-magnon drag effect at *T* = 10 K. To further understand the magnon drag contribution, we plot the difference between $S_{xx}(T)$ measured in a zero and 12 T magnetic field, as shown in inset of Figure 4 (c). The low temperature segment of the plot is well fitted with the $T^{3/2}$ power law, which is expected from the magnon drag effect in the presence of FM-magnons^54^. At temperature above $\sim$ 200 K the behavior of $S_{xx}(T)$ becomes linear, which can be arised from the diffusive contribution. The diffusive contribution to the $S_{xx}(T)$ can be explain by Mott expression^51–53^

6 $$\begin{aligned} \frac{Sxx}{T}= \pm \frac{\pi ^{\ 2}}{2}\frac{k_B}{e}\frac{1}{T_F} =\pm \frac{\pi ^{\ 2}}{3}\frac{k_B^2}{e}\frac{D\left( E_F\right) }{n} \end{aligned}$$

**Fig. 5:**
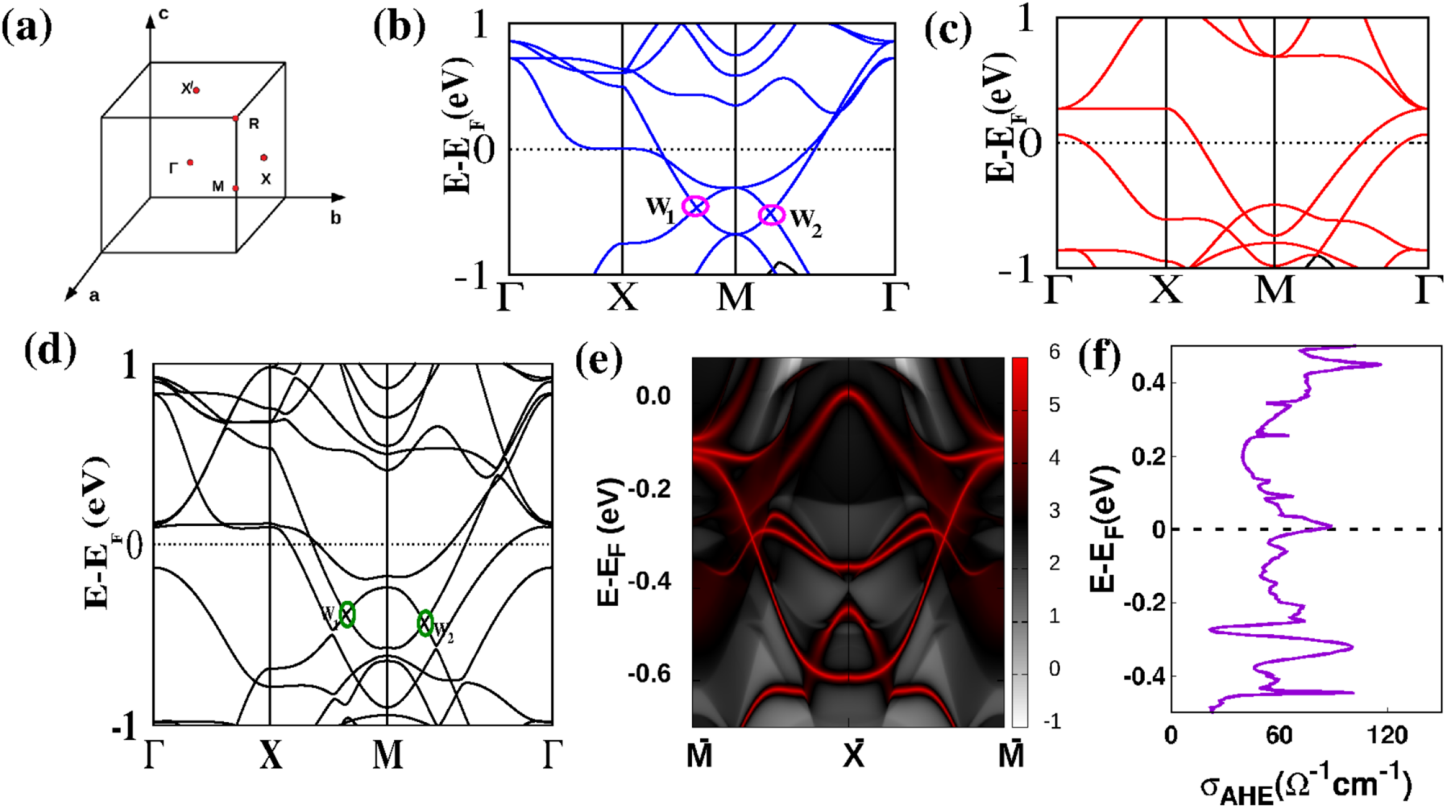
(**a**) High symmetry points are shown in the first Brillouin Zone. (**b**) Band structure for FM configuration calculated with GGA along high symmetry directions for spin down states only (**c**) Band structure for FM configuration calculated with GGA along high symmetry directions for spin up states only. (**d**) Band structure for FM configuration in GGA+SO approximation with Mn moments along [001] axis. (**e**) Plots depicting the spectral function A(**k**;E) in a slab geometry are presented, with **k** denoting momentum within the surface Brillouin zone. Remarkably, distinct red bands appear between the bulk continuum in these plots, revealing the spectrum of surface states. The drumhead-like surface states are highlighted in red, indicative of the presence of Weyl points. (**f**) The calculated value of $\sigma _{AHE}$ at the FL is found to be around 80 $\Omega ^{-1} \text {cm}^{-1}$, which is slightly higher than the experimental value.

where, $k_{B}$, *n*, *e*, $T_{F}$ and $D(\epsilon _{F})$ represent the Boltzmann’s constant, carrier density, electron charge, Fermi temperature and density of states respectively. The density of states is related to Fermi energy as $D(\epsilon _{F})$ = 3n/2$k_{B}\hbox {T}_{{F}}$. Figure 4(d) shows temperature variation of $\frac{S_{xx}}{T}$, the zero extrapolated value of $\frac{S_{xx}}{T}$ is obtained −0.012 $\mu \hbox {VK}^{-1}$, and the corresponding values of Fermi temperature and Fermi energy is found to be $\sim$ 3.45$\times 10^{4}$ K and 2.97 eV respectively. Transport parameters such as Fermi radius ($k_{F}$), Fermi velocity ($v_{F}$) and effective mass (m*) are calculated using zero-temperature limit of $\frac{S_{xx}}{T}$ and given as 1.8$\times$ $10^{7}$ K $\hbox {cm}^{-1}$, 1.1$\times 10^{7}$ m/sec and 0.0088 $m_{e}$ respectively. Behnia $\textit{et al}$. introduced a dimensionless parameter *q* that relates electronic specific heat and the Seebeck coefficient in a single-band approach in the low temperature limit^57^

7 $$\begin{aligned} q = \frac{S_{xx}N_{av}e}{\gamma T} \approx \pm 1 \end{aligned}$$

where, $N_{av}$ and $\gamma$ represent the Avagadro number and Sommerfeld coefficient respectively. The *q* value is found to be 0.035, the positive sign indicates the dominance of hole charge carriers at low temperature in $\hbox {Mn}_{{3}}$GaC. The value of $\gamma$ was calculated from low temperature heat capacity data (see supplementary Figure S5). Furthermore, at low temperature carrier concentration *n* can be calculated using free electron gas approximation

8 $$\begin{aligned} E_F=\frac{\left( hc\right) ^2}{8m_ec^2}\left( \frac{3}{\pi }\ n\right) ^{2/3} \end{aligned}$$

where *h*, *c* and $m_{e}$ represent the Planck’s constant, velocity of light and mass of electron. The estimated value of *n* is obtained $\sim$ $2.2 \times 10^{22} \, \text {cm}^{-3}$, which closely matches with the value obtained from the Hall measurement.

Additionally, we measured the Nernst coefficient ($\nu = \frac{S_{xy}}{H};\quad S_{xy} = \frac{E_y}{|\nabla T_x|}$), which denotes the generation of a transverse electric field when there is a longitudinal temperature gradient and a magnetic field is present^51–53^. Figure 4(e) shows Nernst coefficient $\nu$ as a function of temperature at 2 and 12 T field. The function $\nu (T)$ rises as the temperature increases and exhibits a peak around 50 K, likely attributable to the magnon drag effect. With further increasing the temperature, a broad peak with a value of $\sim$ −0.9 $\mu \hbox {VK}^{-1}\hbox {T}^{-1}$ is observed around 200 K. It is noted that $\nu (T)$ exhibits field independent signature in the intermediate temperature range as plotted by contour graph in Figure 4(f). Although, magnon drag peak shows a clear enhancement with the increase in magnetic field, as expected in literature. The large value of $\nu (T)$ is usually discussed in literature due to non-zero Berry curvature in topological materials such as $\hbox {YbMnSb}_{{2}}$ (3 $\mu \hbox {VK}^{-1}\hbox {T}^{-1}$)^58^ and $\hbox {Co}_{{3}}\hbox {Sn}_{{2}}\hbox {S}_{{2}}$ ($\sim$ 3.8 $\mu$ V $\hbox {K}^{-1}\hbox {T}^{-1}$)^59^. The signature of finite Berry curvature is also found in the AHE of $\hbox {Mn}_{{3}}$GaC. In a single-band picture, $\nu (T)$ is related with the following expression^51–53^

9 $$\begin{aligned} \frac{\nu (T)}{T} = \frac{\pi ^2}{3}\ \frac{k_B}{e}\ \frac{\mu }{T_F} \end{aligned}$$

,where $\hbox {k}_{{B}}$, *e* and $\mu$ represent the Boltzmann constant, electric charge and mobility respectively. This formula establishes a correlation between the Nernst coefficient and carrier mobility. In the zero-temperature limit, the value of $\nu$/T is estimated about $\sim$ −0.0198 $\mu \hbox {VK}^{-2}\hbox {T}^{-1}$ as shown in inset of Figure 4(d) and the corresponding mobility value is found to be $\sim$ 2.5 $\hbox {cm}^{2}$ $\hbox {V}^{-1}\hbox {S}^{-1}$.

### First-principles calculations

Further, we performed electronic band structure calculation on $\hbox {Mn}_{{3}}$GaC. Our total energy calculation considering cubic structure suggests that the FM configuration has lowest energy in comparison to the paramagnetic (PM) and AFM magnetic configurations, indicating its greater stability in the cubic phase as also observed experimentally. Further calculations including spin-orbit interaction were performed to establish the easy axis of magnetization by comparing total energies of the FM configuration with magnetization axis pointing along the [001], [110], and [111] crystallographic axes. We observed [001] direction to be the preferred axis of magnetization.

We have shown the spin-polarized band structure calculated within GGA along high symmetry directions of the BZ shown in Figure 5 (a). There are several Weyl nodes identifiable in the electronic band structure which draws attention to the importance of the FM-semimetallic phase. In Figure 5(b) and (c), we have separately presented down- and up-spin bands respectively. The combined band structure of down- and up-spin is shown in supplementary information (see supplementary Figure S6). Several interesting features are observed in the band structure. A flat spin-up band can be seen extending from $\Gamma$ to X slightly above the FL whereas a flat spin down band can be seen along $\Gamma$-X-M exactly at the FL. Comparing with a sister compound $\hbox {Mn}_3$ZnC^13^ we find that in our system the flat band is present in both spin channels whereas in $\hbox {Mn}_3$ZnC it was found only in the spin-up channel. Several linear band crossings are observed around M and R high symmetry points similar to $\hbox {Mn}_3$ZnC. Two of them along X-M and M-$\Gamma$ directions among the spin-down bands, denoted as W1 and W2 respectively are shown in Figure 5(b). We have investigated these two linear band crossings in the next section. We have plotted density of state (DOS) for non spin polarized and FM configuration calculated with GGA (See supplementary Figure S7).

In the absence of SOC, the band structure shows symmetry-protected crossings in the $k_x = 0$, $k_y = 0$ and $k_z = 0$ planes. These band crossing points are maintained due to the crystal symmetry, resulting in gapless behaviour at specific points in the Brillouin zone (see Figure 5(b),(c) and Figure S8(a), (b)). With SOC included, the band structures for the $k_x = 0$, $k_y = 0$ and $k_z = 0$ planes are significantly different. The SOC interaction leads to the breaking of certain symmetries, especially in the $k_x = 0$ and $k_y = 0$ planes when the moments of the Mn atoms are along [001] direction. Therefore, the band crossings are gapped due to SOC-induced mixing of spin states (see Figure S8 (c)). When SOC is taken into account in $k_z = 0$ plane with moments along [001] direction, due to the preservation of mirror symmetry in the plane ($k_z = 0$) which is not broken by either magnetic ordering or SOC (see Figure 5 (d)), the band crossings remain gapless. Whereas for planes $k_x = 0$ and $k_y = 0$ the mirror symmetry is broken which opens up a gap at the band crossing point along X-M direction as can be seen in Figure S8.

We further calculated the surface states (see Figure 5(e)) by employing a slab geometry to clearly differentiate surface-localized states (highlighted in red) from bulk states (shown in gray). The surface states are distinct from the bulk band structure and remain stable against small perturbations, underscoring their topological protection. These states serve as direct connections between Weyl nodes in the bulk, resulting in gapless states at the Fermi level. Along the $\bar{M} - \bar{X} - \bar{M}$ direction, the surface states cross the Fermi level at specific gapless points. This is a hallmark of materials with Weyl points or other topological characteristics, reflecting a non-trivial topological phase. We have additionally calculated $\sigma _{AHE}$ of $\hbox {Mn}_{{3}}$GaC with respect to the position of FL, depicted in Figure 5(f) for the FM configuration. Our calculated value at FL around 80 $\sim$ 50 $\Omega ^{-1} \text {cm}^{-1}$ is found to be higher than the experimental value but in the same order of magnitude. Notably, the highest peak of the anomalous Hall conductivity that appears around −0.4 eV below the Fermi level, can be attributed to the substantial band dispersion present in that region.

## Summary

In summary, we have presented the evidences of nodal line semimetal in $\hbox {Mn}_{{3}}$GaC through the experimental and band structure calculations. The low-temperature resistivity upturn is well fitted with the Hamann expression, and the corresponding Kondo temperature $T_{K}$ is obtained $\sim$ 16 K. Upon thorough examination of our results, it is evident that AHE in $\hbox {Mn}_{{3}}$GaC is originated by both intrinsic Berry Phase contribution and skew scattering contribution. The anomalous Hall resistivity also demonstrates an upturn at lower temperatures, possibly attributable to the Kondo effect. The low temperature Seebeck data reveals the importance of electron-magnon scattering. A significant large value of the Nernst coefficient reveals the finite Berry curvature of $\hbox {Mn}_{{3}}$GaC compound. The first-principles calculations suggest the existence of the Weyl nodes with contains drumhead surface states, at $\sim$ 0.3 eV below Fermi level, The calculated value of the $\sigma _{AHE}$ is nearly consistent with the experimentally observed value.

## Methods

Polycrystalline samples of $\hbox {Mn}_{{3}}$GaC were prepared in a two-steps process using solid state reaction method. The elements Mn ($\ge$ 99.99%), Ga ($\ge$ 99.99%), and carbon powder (99.9%) were mixed in a 3:1:1 molar ratio and sealed in evacuated ($\sim 10^{-5}$ mbar pressure) quartz tube. In first step, statring material was heated to $800^\circ$C for 192 hours. In second step, pre-reacted compound was grounded, pelletized and again sealed in evacuated quartz tube and heated for 48 hours at $800^\circ$C. The crystal structure and phase purity analysis were determined by powder X-ray diffraction (XRD) using a Rigaku Smart lab diffractometer with Cu-$\hbox {K}_{\alpha }$ radiation ($\lambda$ =1.5418 Å) at room temperature (See supplementary Figure S1). Elemental analysis of the compound using Energy-dispersive X-ray spectroscopy (EDS) and X-ray photoelectron spectroscopy (XPS) techniques (See supplementary Figure S2, Figure S3, Table S1 and Table S2) gave its composition as $\hbox {Mn}_{3.08}\hbox {Ga}_{0.97}\hbox {C}_{0.95}$. The electronic transport properties were measured in the temperature range 1.8–350 K and magnetic field up to 8 T in physical property measurement system (PPMS). The magnetization measurements were performed using a Magnetic Property Measurement System (MPMS). The thermal transport (Seebeck and Nernst effects) measurements were performed on a homemade setup connected with the PPMS^60^.

The electronic band structure calculations were carried out using density functional theory (DFT) calculations, utilizing the Perdew-Burke-Ernzerhof (PBE-GGA) exchange-correlation functional^61^. The computations were performed using the projector-augmented wave (PAW) method^62^ and a plane-wave basis set with the Vienna Ab-initio Simulation Package (VASP)^63,64^. In our self-consistent calculations, a (13x13x13) $\Gamma$-centered Monkhorst-Pack^65^ **k**-point mesh was utilized to integrate the Brillouin zone (BZ). The kinetic energy cutoff for plane waves was set to 600 eV. For the topological property analysis of $\hbox {Mn}_3$GaC, we employed the Wannier90 software package in conjunction with WannierTools^66,67^. Wannier90 utilizes Maximally Localized Wannier Functions (MLWF)^68^ to create a tight-binding model by fitting DFT bands. Subsequently, WannierTools utilizes this tight-binding model to compute various topological properties.

## Supplementary Information

Supplementary Information.

## Data Availability

The data that support the findings of this study are available from the corresponding authors upon reasonable request.

## References

[1] Muechler, L. et al. Modular arithmetic with nodal lines: Drumhead surface states in zrsite. *Phys. Rev. X* **10**, 011026. 10.1103/PhysRevX.10.011026 (2020).

[2] Emmanouilidou, E. et al. Magnetotransport properties of the single-crystalline nodal-line semimetal candidates CaTX (T= Ag, Cd; X = As, Ge). *Phys. Rev. B* **95**, 245113. 10.1103/PhysRevB.95.245113 (2017).

[3] Laha, A. et al. Magnetotransport properties of the topological nodal-line semimetal CaCdSn. *Phys. Rev. B* **102**, 035164. 10.1103/PhysRevB.102.035164 (2020).

[4] Neupane, M. et al. Observation of a three-dimensional topological Dirac semimetal phase in high-mobility . *Nat. communications* **5**, 3786. 10.1038/ncomms4786 (2014). 10.1038/ncomms4786 24807399

[5] Weng, H., Fang, C., Fang, Z., Bernevig, B. A. & Dai, X. Weyl semimetal phase in noncentrosymmetric transition-metal monophosphides. *Phys. Rev. X* **5**, 011029. 10.1103/PhysRevX.5.011029 (2015).

[6] Bian, G. et al. Topological nodal-line fermions in spin-orbit metal . *Nat. communications* **7**, 1–8. 10.1038/ncomms10556 (2016). 10.1038/ncomms10556 PMC4740879 26829889

[7] Shukla, G. K. et al. Anomalous Hall effect from gapped nodal line in the FeGe Heusler compound. *Phys. Rev. B* **104**, 10.1103/PhysRevB.104.195108 (2021).

[8] Obata, Y. et al. ARPES studies of the inverse perovskite PbO: Experimental confirmation of a candidate 3D Dirac fermion system. *Phys. Rev. B* **96**, 10.1103/PhysRevB.96.155109 (2017).

[9] Samal, D., Nakamura, H. & Takagi, H. Molecular beam epitaxy of three-dimensional Dirac material PbO. *APL Materials* **4**, 10.1063/1.4955213 (2016).

[10] Nakamura, H. et al. Robust weak antilocalization due to spin-orbital entanglement in Dirac material SnO. *Nat. communications* **11**, 1161. 10.1038/s41467-020-14900-1 (2020). 10.1038/s41467-020-14900-1 PMC7054336 32127524

[11] Yu, R., Weng, H., Fang, Z., Dai, X. & Hu, X. Topological node-line semimetal and Dirac semimetal state in antiperovskite PdN. *Phys. review letters* **115**, 10.1103/PhysRevLett.115.036807 (2015). 10.1103/PhysRevLett.115.036807 26230820

[12] Zhou, X. et al. Spin-order dependent anomalous Hall effect and magneto-optical effect in the noncollinear antiferromagnets XN with X = Ga, Zn, A, or Ni. *Phys. Rev. B* **99**, 10.1103/PhysRevB.99.104428 (2019).

[13] Teicher, S. M., Svenningsson, I. K., Schoop, L. M. & Seshadri, R. Weyl nodes and magnetostructural instability in antiperovskite ZnC. *APL Mater.* **7**, 10.1063/1.5129689 2019).

[14] Wen, Y. et al. Influence of carbon content on the lattice variation, magnetic and electronic transport properties in . *Appl. Phys. Lett. ***96**, 10.1063/1.3295695 (2010).

[15] Lewis, L. H., Yoder, D., Moodenbaugh, A., Fischer, D. A. & Yu, M. Magnetism and the defect state in the magnetocaloric antiperovskite . *J. Physics: Condens. Matter* **18**, 1677. 10.1088/0953-8984/18/5/020 (2006).

[16] Dias, E., Priolkar, K. & Nigam, A. Effect of carbon content on magnetostructural properties of GaC. *J. magnetism and magnetic materials* **363**, 140–144. 10.1016/j.jmmm.2014.03.052 (2014).

[17] Dias, E., Priolkar, K., Ranjan, R., Nigam, A. & Emura, S. Mechanism of magnetostructural transformation in multifunctional GaC. *J. Appl. Phys. ***122**, 10.1063/1.4996933 (2017).

[18] Dias, E. et al. Effect of local structural distortions on magnetostructural transformation in SnC. *J. Phys. D: Appl. Phys.* **48**, 10.1088/0022-3727/48/29/295001 (2015).

[19] Brockhouse, B. & Myers, H. New type of magnetic transition in ZnC. *Can. J. Phys.* **35**, 313–323. 10.1139/p57-035 (1957).

[20] Kim, I. G., Jin, Y. J., Lee, J. I. & Freeman, A. Spin-density inversion symmetry driven first-order magnetic phase transition in . *Phys. Rev. B* **67**, 10.1103/PhysRevB.67.060407 (2003).

[21] Swanson, M. & Friedberg, S. The electrical resistivity of ZnC between and K. *Can. J. Phys.* **39**, 1429–1432. 10.1139/p61-171 (1961).

[22] Barua, S., Hatnean, M. C., Lees, M. R. & Balakrishnan, G. Signatures of the Kondo effect in . *Sci. reports* **7**, 10964. 10.1038/s41598-017-11247-4 (2017). 10.1038/s41598-017-11247-4 PMC5591204 28887567

[23] Maritato, L. et al. Low-temperature resistivity of ultra thin films: Role of quantum interference effects. *Phys. Rev. B* **73**, 10.1103/PhysRevB.73.094456 (2006).

[24] Biswas, D., Meikap, A., Chattopadhyay, S., Chatterjee, S. & Lin, J.-J. Weak localization and electron-electron interaction in disordered alloys at low temperature. *Phys. Lett. A* **328**, 380–386. 10.1016/j.physleta.2004.06.016 (2004).

[25] Han, Y.-L. et al. Carrier-mediated Kondo effect and Hall mobility by electrolyte gating in slightly doped anatase films. *Phys. Rev. B* **90**, 10.1103/PhysRevB.90.205107 (2014).

[26] Wang, Y. et al. Weak Kondo effect in the monocrystalline transition metal dichalcogenide . *Phys. Rev. B* **103**, 10.1103/PhysRevB.103.174418 (2021).

[27] Nobukane, H., Tabata, Y., Kurosawa, T., Sakabe, D. & Tanda, S. Coexistence of the Kondo effect and spin glass physics in Fe-doped . *J. physics: condensed matter* **32**, 10.1088/1361-648X/ab622a (2020). 10.1088/1361-648X/ab622a 31842001

[28] Bakanowski, S., Crow, J. & Mihalisin, T. Kondo scattering from Sm ions in . *Solid State Commun.* **22**, 241–244. 10.1016/0038-1098(77)90402-1 (1977).

[29] Mott, N. *Metal-insulator transitions* (CRC Press, 2004).

[30] Chatterjee, S. et al. Nodal-line and triple point fermion induced anomalous Hall effect in the topological Heusler compound CrGa. *Phys. Rev. B* **107**, 10.1103/PhysRevB.107.125138 (2023).

[31] Bera, S. et al. Anomalous Hall effect induced by Berry curvature in the topological nodal-line van der Waals ferromagnet . *Phys. Rev. B* **108**, 115122. 10.1103/PhysRevB.108.115122 (2023).

[32] Nagaosa, N., Sinova, J., Onoda, S., MacDonald, A. H. & Ong, N. P. Anomalous hall effect. *Rev. Mod. Phys.* **82**, 1539. 10.1103/RevModPhys.82.1539 (2010).

[33] Liu, E. et al. Giant anomalous hall effect in a ferromagnetic kagome-lattice semimetal. *Nat. Phys.* **14**, 1125–1131. 10.1038/s41567-018-0234-5 (2018). 30416534 10.1038/s41567-018-0234-5 PMC6217931

[34] Siddiquee, H. et al. Breakdown of the scaling relation of anomalous hall effect in Kondo lattice ferromagnet USbTe. *Nat. Commun.* **14**, 527. 10.1038/s41467-023-36221-9 (2023). 36720874 10.1038/s41467-023-36221-9 PMC9889341

[35] Shahi, N. et al. Antisite disorder and Berry curvature driven anomalous Hall effect in the spin gapless semiconducting CoAl Heusler compound. *Phys. Rev. B* **106**, 10.1103/PhysRevB.106.245137 (2022).

[36] Yu, Z. et al. Large anomalous hall effect induced by skew scattering in the hexagonal ferromagnet PrCrGe_3_. *Phys. Rev. B* **111**, 125112. 10.1103/PhysRevB.111.125112 (2025).

[37] Kan, D., Xie, L. & Shimakawa, Y. Scaling of the anomalous Hall effect in perpendicularly magnetized epitaxial films of the ferrimagnet . *Phys. Rev. B* **104**, 10.1103/PhysRevB.104.134407 (2021).

[38] Dulal, R. P. et al. Weak localization and small anomalous Hall conductivity in ferromagnetic Weyl semimetal TiGe. *Sci. reports* **9**, 3342. 10.1038/s41598-019-39037-0 (2019). 10.1038/s41598-019-39037-0 PMC6399263 30833580

[39] Nakatsuji, S., Kiyohara, N. & Higo, T. Large anomalous hall effect in a non-collinear antiferromagnet at room temperature. *Nature* **527**, 212–215. 10.1038/nature15723 (2015). 26524519 10.1038/nature15723

[40] Imort, I.-M., Thomas, P., Reiss, G. & Thomas, A. Anomalous Hall effect in the Co-based Heusler compounds FeSi and FeAl. *J. Appl. Phys.* **111**, 10.1063/1.3678323 (2012).

[41] Manyala, N. et al. Large anomalous hall effect in a silicon-based magnetic semiconductor. *Nat. Mater.* **3**, 255–262. 10.1038/nmat1103 (2004). 15034565 10.1038/nmat1103

[42] Fang, Z. et al. The anomalous hall effect and magnetic monopoles in momentum space. *Science* **302**, 92–95. 10.1126/science.1089408 (2003). 14526076 10.1126/science.1089408

[43] Kim, T., Lim, S. & Gambino, R. Spontaneous Hall effect in amorphous and Sm-Fe thin films. *J. Appl. Phys.* **89**, 7212–7214. 10.1063/1.1357117 (2001).

[44] Dijkstra, J., Zijlema, P., Van Bruggen, C., Haas, C. & De Groot, R. Band-structure calculations of and , and transport and magnetic properties of . *J. Physics: Condensed Matter* **1**, 6363. 10.1088/0953-8984/1/36/005 (1989).

[45] Nayak, A. K. et al. Large anomalous Hall effect driven by a nonvanishing Berry curvature in the noncolinear antiferromagnet Mn3Ge. *Sci. advances* **2**, (2016). 10.1126/sciadv.1501870 PMC4846447 27152355

[46] Miyasato, T. et al. Crossover behavior of the anomalous Hall effect and anomalous Nernst effect in itinerant ferromagnets. *Phys. Rev. Lett.* **99**, 086602. 10.1103/PhysRevLett.99.086602 (2007). 17930968 10.1103/PhysRevLett.99.086602

[47] Kim, T. & Gambino, R. Composition dependence of the Hall effect in amorphous thin films. *J. Appl. Phys.* **87**, 1869–1873. 10.1063/1.372106 (2000).

[48] Ye, L. et al. Massive Dirac fermions in a ferromagnetic kagome metal. *Nature* **555**, 638–642. 10.1038/nature25987 (2018). 29555992 10.1038/nature25987

[49] Yu, J. et al. Magnetotransport and magnetic properties of molecular-beam epitaxy L10 FePt thin films. *J. Appl. Phys.* **87**, 6854–6856. 10.1063/1.372864 (2000).

[50] Matusiak, M., Plackowski, T., Bukowski, Z., Zhigadlo, N. & Karpinski, J. Evidence of spin-density-wave order in from measurements of thermoelectric power. *Phys. Rev. B* **79**, 10.1103/PhysRevB.79.212502 (2009).

[51] Gangwar, S. et al. Chiral anomaly and positive longitudinal magnetoresistance in the type-II Dirac semimetals (A= Cu, Ag). *Phys. Rev. B* **108**, 10.1103/PhysRevB.108.245141 (2023).

[52] Sharma, S., Kumar, S., Kumar, A., Shimada, K. & Yadav, C. S. Electronic transport studies of -doped topological insulator. *J. Appl. Phys.* **132**, 10.1063/5.0102131 (2022).

[53] Sharma, S. & Yadav, C. S. Anisotropic upper critical field, Seebeck and Nernst coefficient of superconductor. *Supercond. Sci. Technol.* **35**, 10.1088/1361-6668/ac74e9 (2022).

[54] Pallecchi, I., Caglieris, F. & Putti, M. Thermoelectric properties of iron-based superconductors and parent compounds. *Supercond. Sci. Technol.* **29**, 073002. 10.1088/0953-2048/29/7/073002 (2016).

[55] Banerjee, A., Pal, S., Bhattacharya, S., Chaudhuri, B. & Yang, H. Magnetoresistance and magnetothermoelectric power of . *Phys. Rev. B* **64**, 10.1103/PhysRevB.64.104428 (2001).

[56] Mandal, P. Temperature and doping dependence of the thermopower in . *Phys. Rev. B* **61**, 14675. 10.1088/0953-8984/16/28/037 (2000).

[57] Behnia, K., Jaccard, D. & Flouquet, J. On the thermoelectricity of correlated electrons in the zero-temperature limit. *J. Phys. Condens. Matter* **16**, 5187. 10.1088/0953-8984/16/28/037 (2004).

[58] Xu, S. et al. Large Nernst effect and possible temperature-induced Lifshitz transition in topological semimetal . *Phys. Rev. B* **107**, 10.1103/PhysRevB.107.245138 (2023).

[59] Ding, L. et al. Intrinsic anomalous Nernst effect amplified by disorder in a half-metallic semimetal. *Phys. Rev. X* **9**, 041061. 10.1103/PhysRevX.9.041061 (2019).

[60] Sharma, S. & Yadav, C. S. Experimental setup for the Seebeck and Nernst coefficient measurements. *Rev. Sci. Instrum.*10.1063/5.0031544 (2020). 33380004 10.1063/5.0031544

[61] Perdew, J. P. et al. Restoring the density-gradient expansion for exchange in solids and surfaces. *Phys. Rev. Lett.* **100**, 136406. 10.1103/PhysRevLett.100.136406 (2008). 18517979 10.1103/PhysRevLett.100.136406

[62] Blöchl, P. E. Projector augmented-wave method. *Phys. Rev. B* **50**, 17953. 10.1103/PhysRevB.50.17953 (1994). 10.1103/physrevb.50.17953 9976227

[63] Kresse, G. & Furthmüller, J. Efficient iterative schemes for ab initio total-energy calculations using a plane-wave basis set. *Phys. Rev. B* **54**, 11169. 10.1103/PhysRevB.54.11169 (1996). 10.1103/physrevb.54.11169 9984901

[64] Kresse, G. & Furthmüller, J. Efficiency of ab-initio total energy calculations for metals and semiconductors using a plane-wave basis set. *Comput. Mater. Sci.* **6**, 15–50. 10.1016/0927-0256(96)00008-0 (1996). 10.1103/physrevb.54.11169 9984901

[65] Special points for Brillouin-zone integrations, author=Monkhorst, Hendrik J and Pack, James D. *Phys. review B* **13**, 5188, 10.1103/PhysRevB.13.5188 (1976).

[66] Wu, Q., Zhang, S., Song, H.-F., Troyer, M. & Soluyanov, A. A. WannierTools: An open-source software package for novel topological materials. *Comput. Phys. Commun.* **224**, 405–416. 10.1016/j.cpc.2017.09.033 (2018).

[67] Mostofi, A. A. et al. An updated version of wannier90: A tool for obtaining maximally-localised wannier functions. *Comput. Phys. Commun.* **185**, 2309–2310. 10.1016/j.cpc.2014.05.003 (2014).

[68] Marzari, N., Mostofi, A. A., Yates, J. R., Souza, I. & Vanderbilt, D. Maximally localized wannier functions: Theory and applications. *Rev. Mod. Phys.* **84**, 1419. 10.1103/RevModPhys.84.1419 (2012).

