# Supplementary material for: Evidences of topological nodal line semimetal in Mn3GaC: Anomalous Hall effect, thermal transport and DFT studies
Source: Sci Rep. 2025 Sep 29;15:33639. doi: 10.1038/s41598-025-10563-4 (PMC12480682; doi:10.1038/s41598-025-10563-4)
Supplement: Supplementary file 1 — Supplementary Information. [file 41598_2025_10563_MOESM1_ESM.docx]

**Supplementary Information**

**Evidences of topological nodal line semimetal in Mn_3_GaC: Anomalous Hall effect,**

**thermal transport and DFT studies**

Sunil Gangwar^1^, Amarjyoti Choudhury^2^, Tulika Maitra^2^, and C. S. Yadav^1∗^

School of Physical Sciences, Indian Institute of Technology Mandi, Kamand, Mandi-175075 (H.P.) India and Department of Physics, Indian Institute of Technology Roorkee, Uttrakhand-247667, India

**X- ray diffraction:**


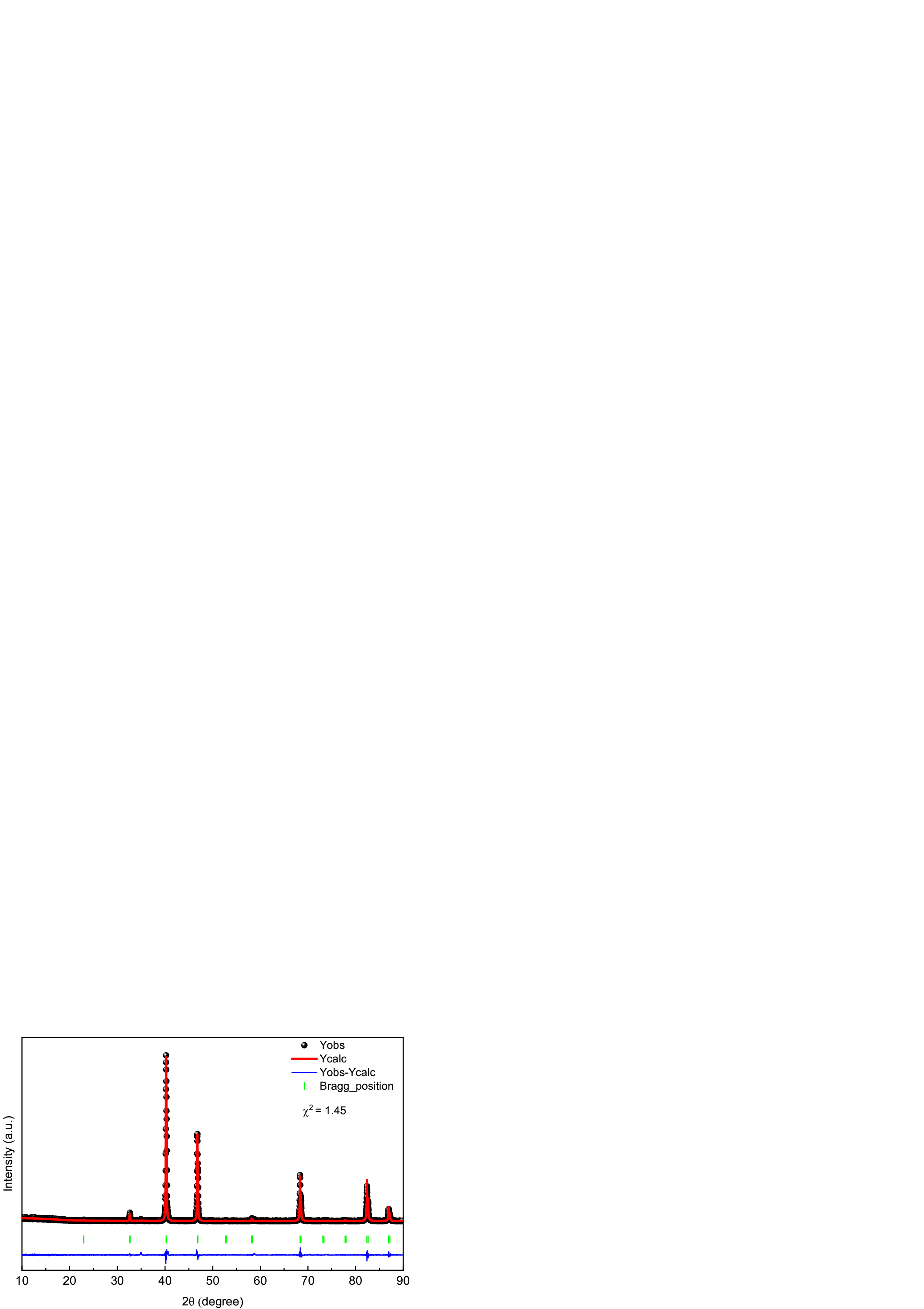


Figure S1. Powder x–ray diffraction pattern showing the clean single phase (*Pm-3m*).

Figure S1 presents the X-ray powder diffraction pattern along with its corresponding Rietveld refinement. The Rietveld refinement of the XRD data confirms that the sample is formed in a single phase. The refined lattice parameters calculated from the Rietveld fit are a = b = c = 3.8794 Å. The atoms Mn, Ga and C within the Mn_3_GaC are positioned at the Wyckoff coordinates 3*c* (0, 0.5, 0.5), 1*a* (0, 0, 0), and 1*b* (0.5, 0.5, 0.5), respectively.

**X-ray photoelectron spectroscopy (XPS):**

All the elements have detectable XPS signals in the spectrum, proving their existence and revealing information about their chemical states and binding environments. The analyzed XPS data provides information on the atomic percentage of elements in Mn_3_GaC, as shown in Table 1. Mn_3_GaC exhibits deficiencies in both Ga and C, resulting in a final atomic composition where Mn is present at 3.10 atomic percent, Ga at 0.97 atomic percent, and C at 0.93 atomic percent (Mn_3.10_Ga_0.97_C_0.93_).

**Supplementary Table S1:** **Representative elemental compositions of the Mn_3_GaC Obtained Using XPS analysis**

| **Elements** | **Peak BE** | **FWHM (eV)** | **Area (eV)** | **Atomic %** | **Error** | **Q** | **SF** |
| --- | --- | --- | --- | --- | --- | --- | --- |
| Mn 2p | 638.15 | 1.65 | 601657.12 | 62.05 | ± 3.10 | 1 | 12.353 |
| Ga 3d | 17.6 | 1.25 | 32113.91 | 19.33 | ± 0.967 | 1 | 1.412 |
| C 1s | 282.2 | 1.05 | 17884.66 | 18.62 | ± 0.931 | 1 | 1 |


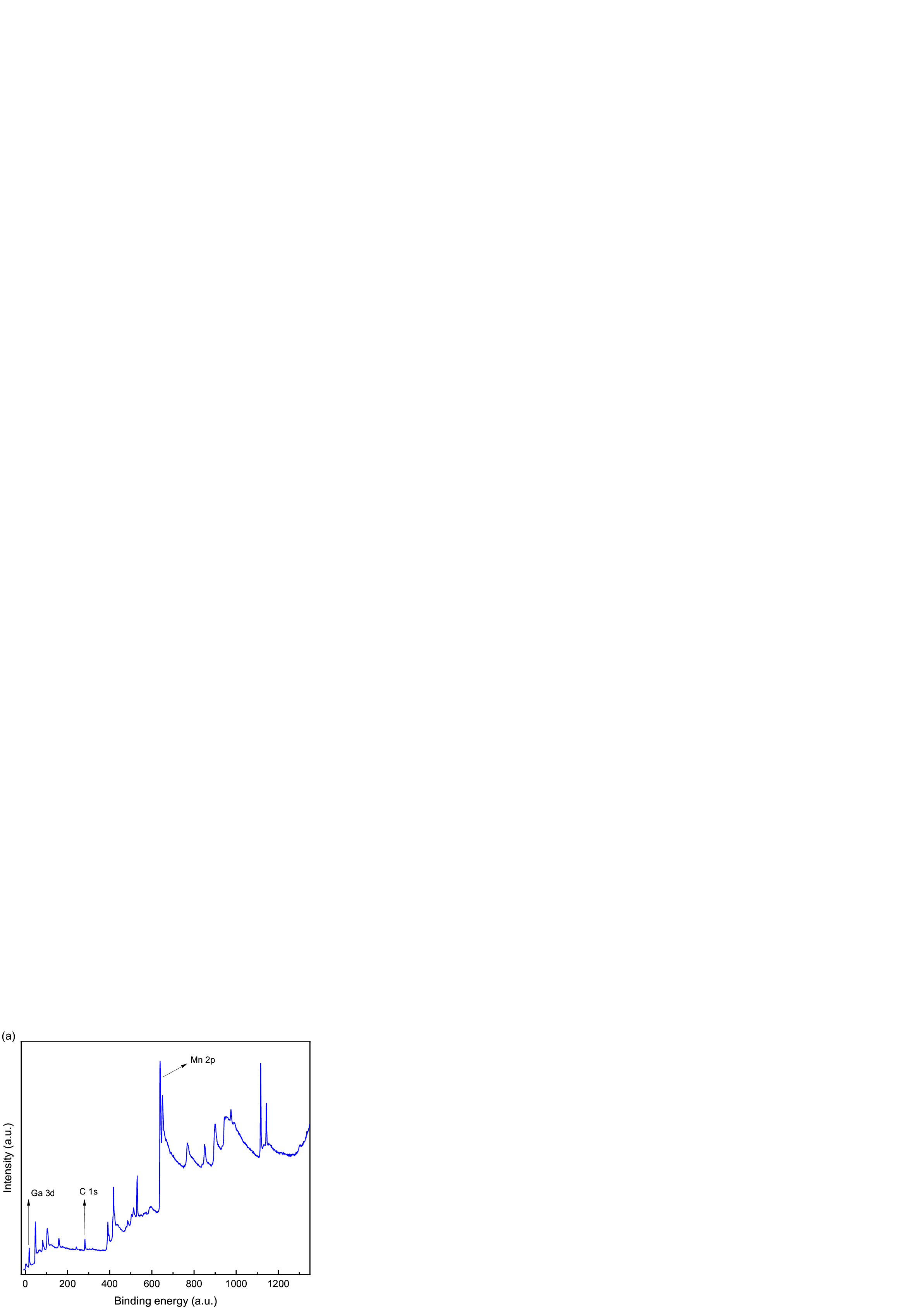


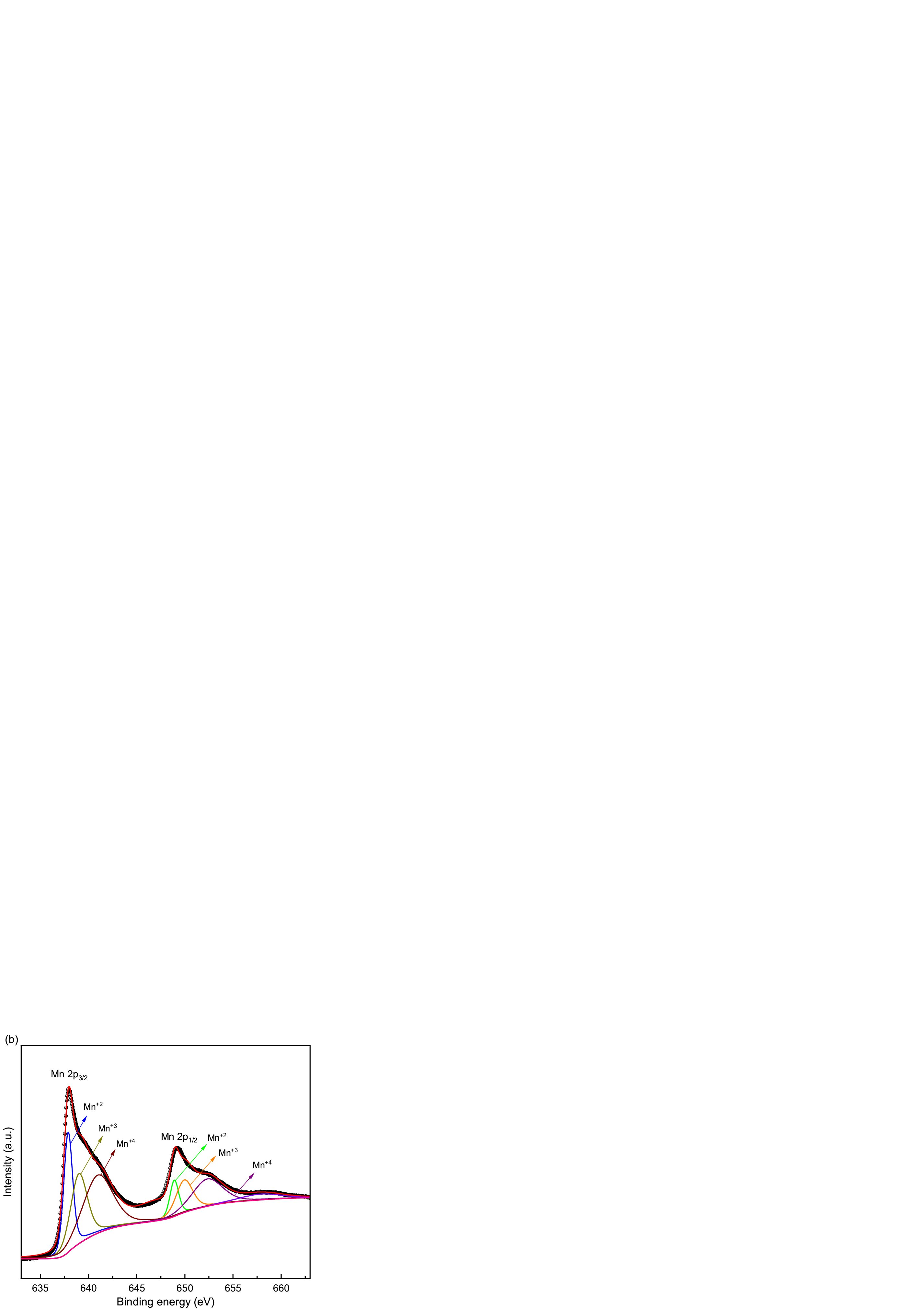


Figure S2 (a) X-Ray Photoelectron Spectroscopy survey spectrum for Mn_3_GaC. (b) XPS spectra of Mn 2p core-level.

It is confirmed from the XPS data fitting of Mn 2p that Mn^+2^ ions have the maximum intensity in XPS spectra. Thus, Mn^+2^ ions play a dominant role in the origin of the Kondo effect in Mn_3_GaC.

**Energy dispersive spectrum:**

We have performed Energy dispersive spectrum (EDS) measurement to determine elementary compostion of Mn_3_GaC compound. Figure S4 shows scanning electron microscopy (SEM) image at 8 different spots to obtain accurate elemental composition. We present EDS spectra only for a one spot in Figure S5.


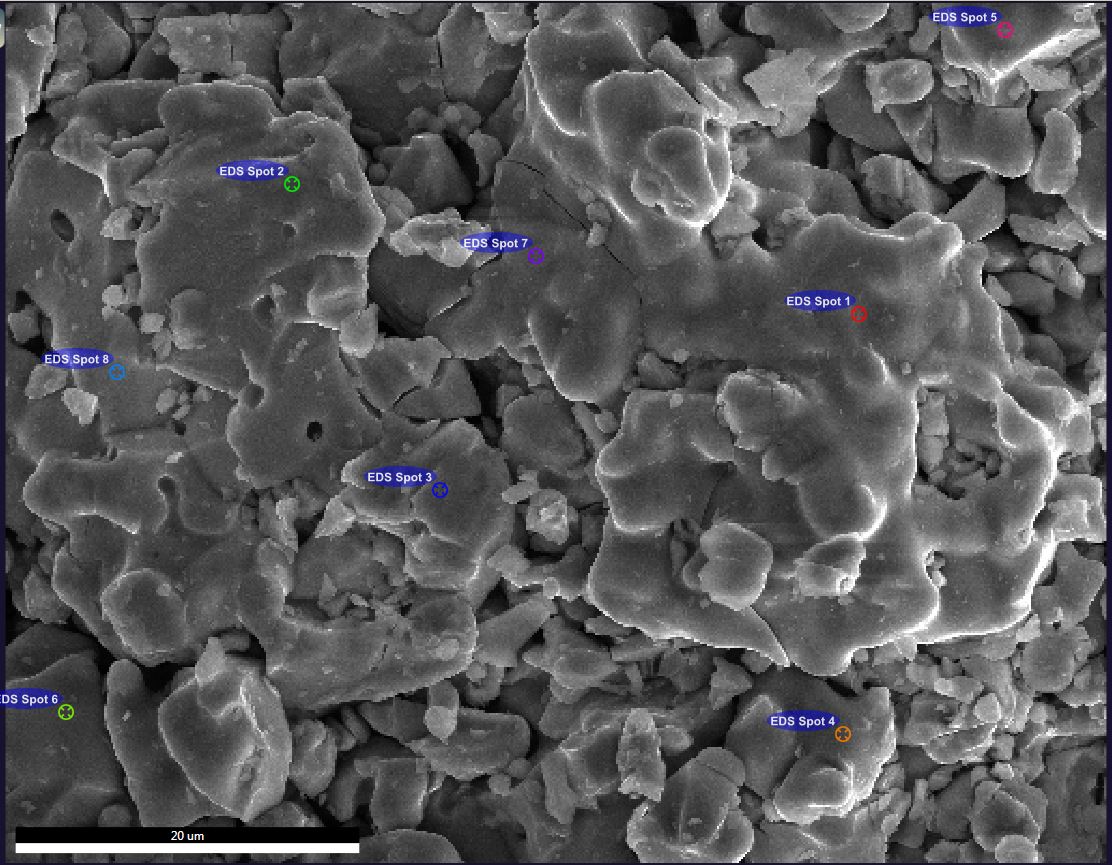


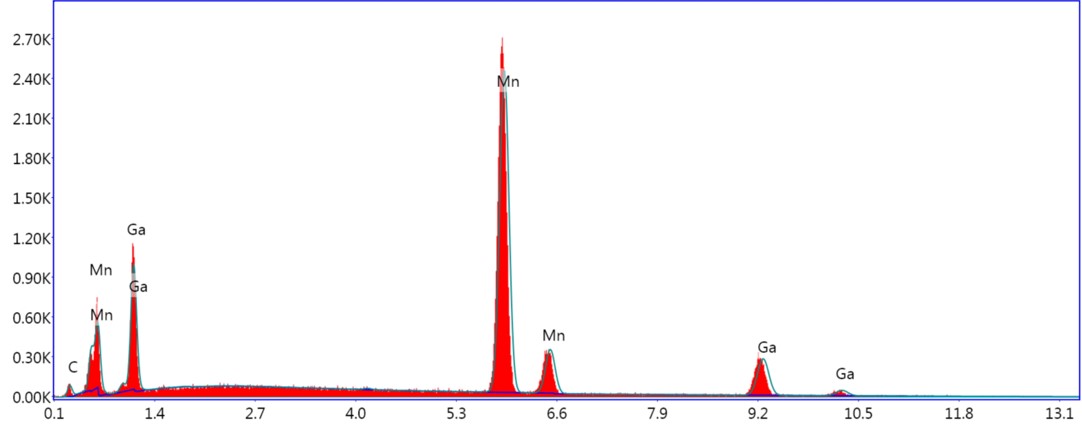


Figure S3 (a) SEM image of 8 – different spots for Mn_3_GaC polycrystal. (b) EDS spectra showing the peaks of constituent elements of Mn_3_GaC polycrystal crystal

**Supplementary Table S2: Representative elemental compositions of the Mn_3_GaC Obtained Using EDS analysis**

Spot:1

| Element | Weight % | Atomic % | Net Int. | Error % | Z | R | A | F |
| --- | --- | --- | --- | --- | --- | --- | --- | --- |
| C K | 4.96 | 20.28 | 17.78 | 16.48 | 1.34 | 0.84 | 0.19 | 1 |
| MnK | 67.82 | 60.57 | 1,226.77 | 2.03 | 0.99 | 1 | 0.99 | 1.02 |
| GaK | 27.21 | 19.15 | 163.71 | 5.03 | 0.94 | 1.02 | 0.96 | 1 |

Spot: 2

| Element | Weight % | Atomic % | Net Int. | Error % | Z | R | A | F |
| --- | --- | --- | --- | --- | --- | --- | --- | --- |
| C K | 5.59 | 22.40 | 19.74 | 16.27 | 1.33 | 0.84 | 0.19 | 1 |
| MnK | 66.83 | 58.55 | 1,192.33 | 2.03 | 0.99 | 1 | 0.99 | 1.02 |
| GaK | 27.58 | 19.04 | 163.69 | 4.99 | 0.94 | 1.02 | 0.96 | 1 |

Spot: 3

| Element | Weight % | Atomic % | Net Int. | Error % | Z | R | A | F |
| --- | --- | --- | --- | --- | --- | --- | --- | --- |
| C K | 4.71 | 19.41 | 16.16 | 16.66 | 1.34 | 0.84 | 0.19 | 1 |
| MnK | 67.67 | 60.97 | 1,179.43 | 2.00 | 1 | 1 | 0.99 | 1.02 |
| GaK | 27.62 | 19.61 | 160.14 | 4.77 | 0.94 | 1.02 | 0.96 | 1 |

Spot: 4

| Element | Weight % | Atomic % | Net Int. | Error % | Z | R | A | F |
| --- | --- | --- | --- | --- | --- | --- | --- | --- |
| C K | 4.10 | 17.23 | 14.69 | 18.23 | 1.34 | 0.84 | 0.19 | 1 |
| MnK | 68.74 | 63.11 | 1,248.15 | 2.02 | 1 | 1 | 0.99 | 1.02 |
| GaK | 27.16 | 19.65 | 164.05 | 5.04 | 0.94 | 1.02 | 0.96 | 1 |

Spot: 5

| Element | Weight % | Atomic % | Net Int. | Error % | Z | R | A | F |
| --- | --- | --- | --- | --- | --- | --- | --- | --- |
| C K | 4.73 | 19.47 | 16.90 | 16.57 | 1.34 | 0.84 | 0.19 | 1 |
| MnK | 67.71 | 60.98 | 1,229.08 | 2.03 | 1 | 1 | 0.99 | 1.02 |
| GaK | 27.56 | 19.56 | 166.38 | 4.95 | 0.94 | 1.02 | 0.96 | 1 |

Spot: 6

| Element | Weight % | Atomic % | Net Int. | Error % | Z | R | A | F |
| --- | --- | --- | --- | --- | --- | --- | --- | --- |
| C K | 4.41 | 18.36 | 15.64 | 17.96 | 1.34 | 0.84 | 0.19 | 1 |
| MnK | 67.97 | 61.84 | 1,225.91 | 2.03 | 1 | 1 | 0.99 | 1.02 |
| GaK | 27.62 | 19.80 | 165.72 | 4.84 | 0.94 | 1.02 | 0.96 | 1 |

Spot: 7

| Element | Weight % | Atomic % | Net Int. | Error % | Z | R | A | F |
| --- | --- | --- | --- | --- | --- | --- | --- | --- |
| C K | 5.33 | 21.53 | 19.37 | 16.41 | 1.33 | 0.84 | 0.19 | 1 |
| MnK | 67.12 | 59.29 | 1,234.07 | 2.02 | 0.99 | 1 | 0.99 | 1.02 |
| GaK | 27.55 | 19.18 | 168.49 | 5.09 | 0.94 | 1.02 | 0.96 | 1 |

Spot: 8

| Element | Weight % | | Atomic % | Net Int. | Error % | Z | R | A | F |
| --- | --- | --- | --- | --- | --- | --- | --- | --- | --- |
| C K | 4.22 | 17.69 | | 14.20 | 18.28 | 1.34 | 0.84 | 0.19 | 1 |
| MnK | 67.41 | 61.81 | | 1,163.88 | 2.01 | 1 | 1 | 0.99 | 1.02 |
| GaK | 28.37 | 20.50 | | 162.94 | 4.72 | 0.94 | 1.02 | 0.96 | 1 |

We average the elemental compositions across various spots and the final composition is found to be Mn_3.08_Ga_0.97_C_0.95_.

**Supplementary Table 3. Summary of anomalous Hall conductivity (*σ_AHE_*), and anomalous Hall angle (*θ_A_*) of currently studied magnetic materials.**

| **Materials** | ***σ_AHE_* (Ω^-1^cm-1)** | ***θ_A_* %** | **References** |
| --- | --- | --- | --- |
| Mn_3_Sn | 100 | 1.1 | 1 |
| Co_2_FeAl | 120 | 1.3 | 2 |
| MnSi | 150 | 3.7 | 3 |
| SrRuO_3_ film | 200 | 1 | 4 |
| Co_2_FeSi | 208 | 1.8 | 2 |
| SmFe film | 317 | 4.8 | 5 |
| Fe_0.28_TaS_2_ | 336 | 3.7 | 6 |
| Mn_3_Ge | 450 | 5 | 7 |
| CuZnCrSe | 500 | 1 | 8 |
| TbCo film | 800 | 3.2 | 9 |
| Fe_3_Sn_2_ | 1100 | 1.1 | 10 |
| Fe film | 1134 | 2.6 | 8 |
| L10-FePt | 1250 | 3.3 | 11 |
| LaSrCoO | 100 | 1 | 8 |
| Ni thin film | 800 | 0.8 | 8 |
| Gd thin film | 1000 | 2 | 8 |
| Mn_3_GaC | 75 | 1.1 | **Present work** |

**Longitudinal resistivity vs. applied magnetic field**

We measured the longitudinal resistivity (*ρ_xx_*) as a function of applied magnetic field in the temperature range of 1.8 – 300 K, as shown in fig. S8.


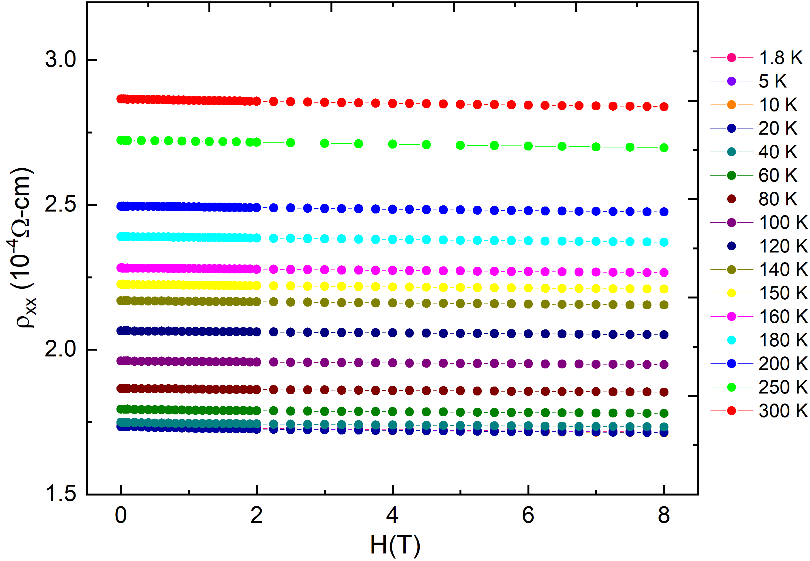


Figure S4: longitudinal resistivity (*ρ_xx_*) as a function of applied magnetic field at different temperatures.

**Heat capacity:**

In general, specific heat of magnetic material contain contributions from electronic magnon and phonon and can be expressed as *C*_total_ = *C*_elec_ + *C*_lattice_ + *C*_magnon,_ where *C*_elec_, *C*_magnon_ and *C*_lattice_ represent the electronic contribution varies as *aT*, magnon contribution varies as *bT^3/2^* and lattice contribution varies as *cT^3^*, respectively. The fitting parameter are found to be a = 0.038 J/mol-K^2^, b = 0.000176 J/mol-K^5/2^ and c = 0.00337 J/mol-K^4^. Using the fitted parameters, we plot the individual contributions to the heat capacity, clearly showing that the lattice contribution dominant but magnon contribution also plays a significant role. We have included this analysis in the revised supplementary information.


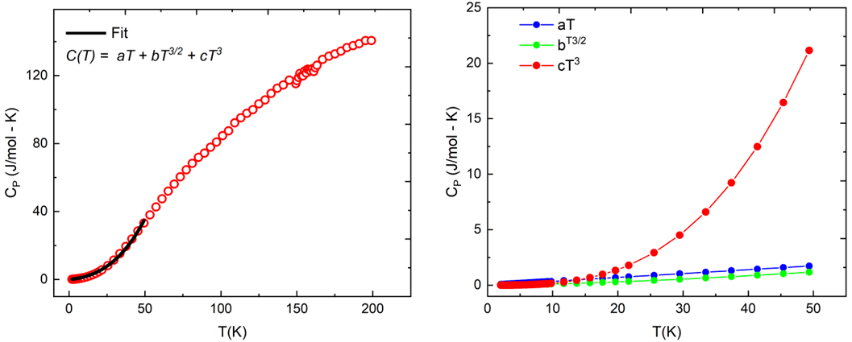

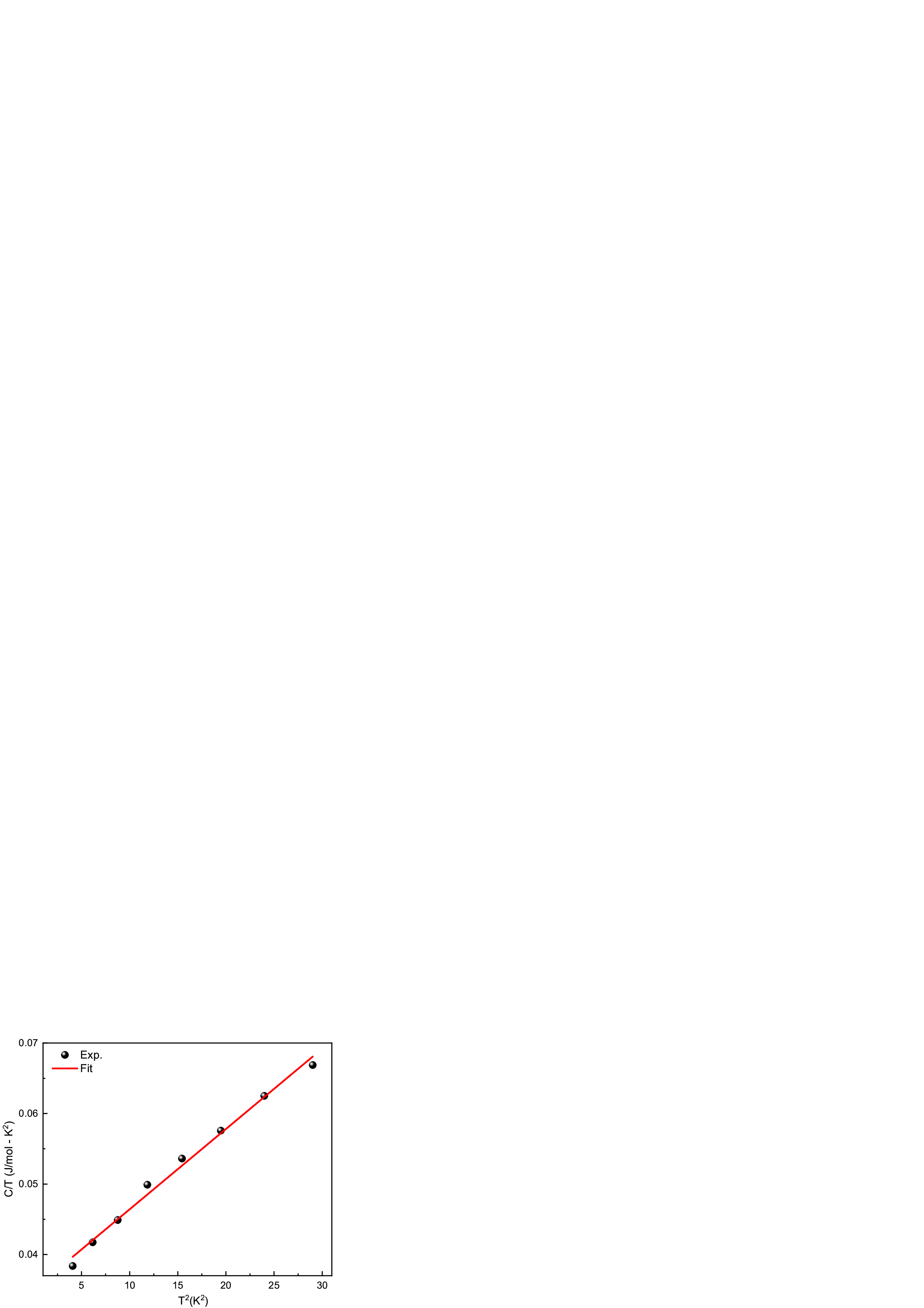


Figure S5: Left: Heat capacity as a function temperature and solid blcak line represents the fitting using the expression C(T) = *aT* + *bT^3/2^* + *cT^3^*. Middle: Different contribution (electronic, mogon and lattice contribution) as a fuction of temperature. C/T versus T^2^ plot. The low-temperature data are fitted using the equation 𝐶⁄𝑇 = 𝛾 + 𝛽𝑇^2^

The low temperature heat capacity of normal metal has electronic and contribution (*γT*) and phonon contribution (*βT^3^*) ascribed to the free electron and lattice vibration respectively. The total heat capacity formula is given by –

*C* (*T*) = *γT* + *βT^3^*

Figure S6 shows the variation of *C/T* with *T*^2^ in low temperature region. The coefficients linked with these terms are calculated by fitting the plot of *C*/*T* vs *T^2^* with a linear equation. The intercept and slope of the linear fit provide the values of γ and β, respectively. The obtained values of γ and β are found to be 35 mJ/mol-K^2^ and 0.0014 mJ/mol-K^4^ respectively.


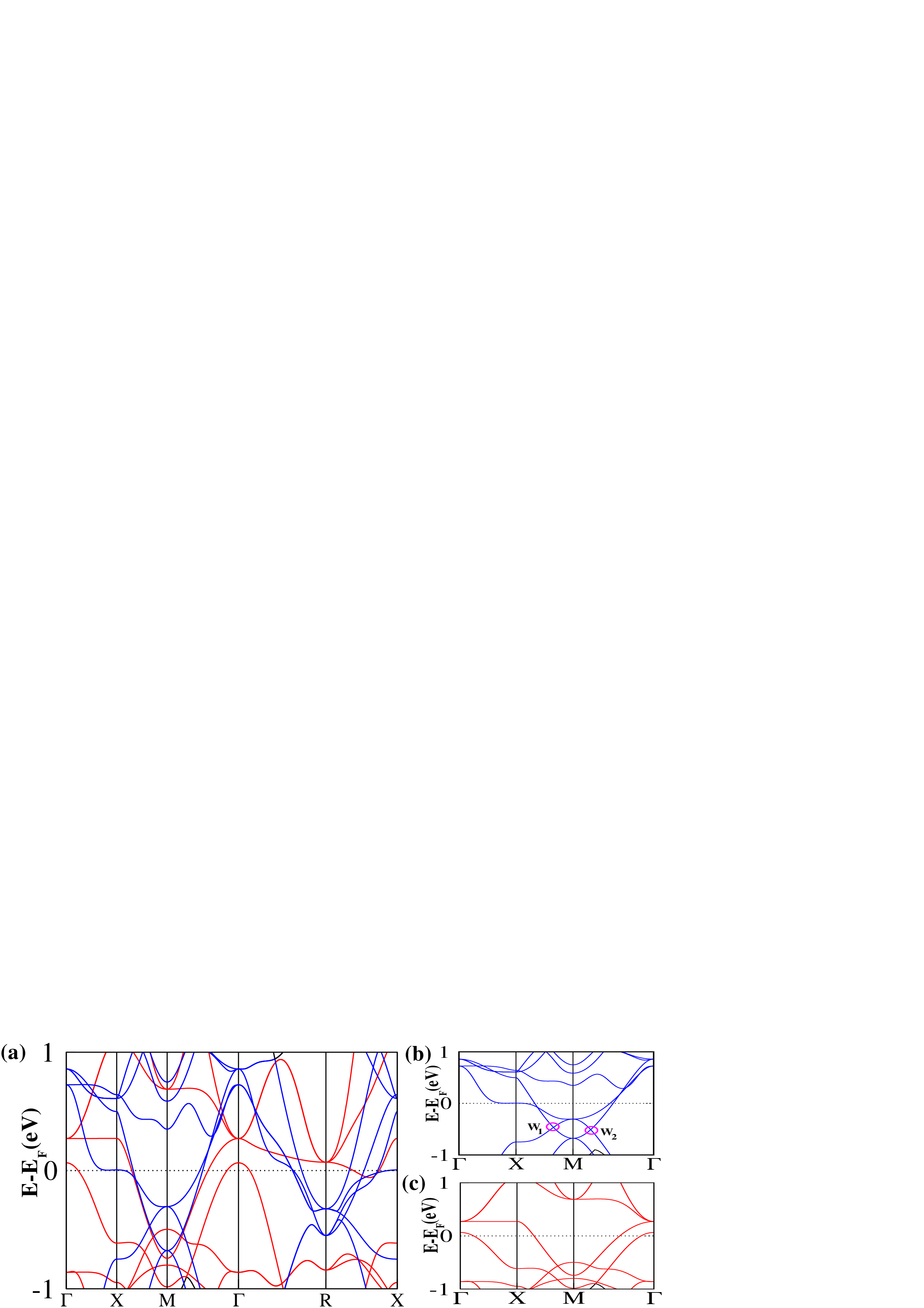


Figure S6. Band structure for FM configuration calculated with GGA along high symmetry directions.


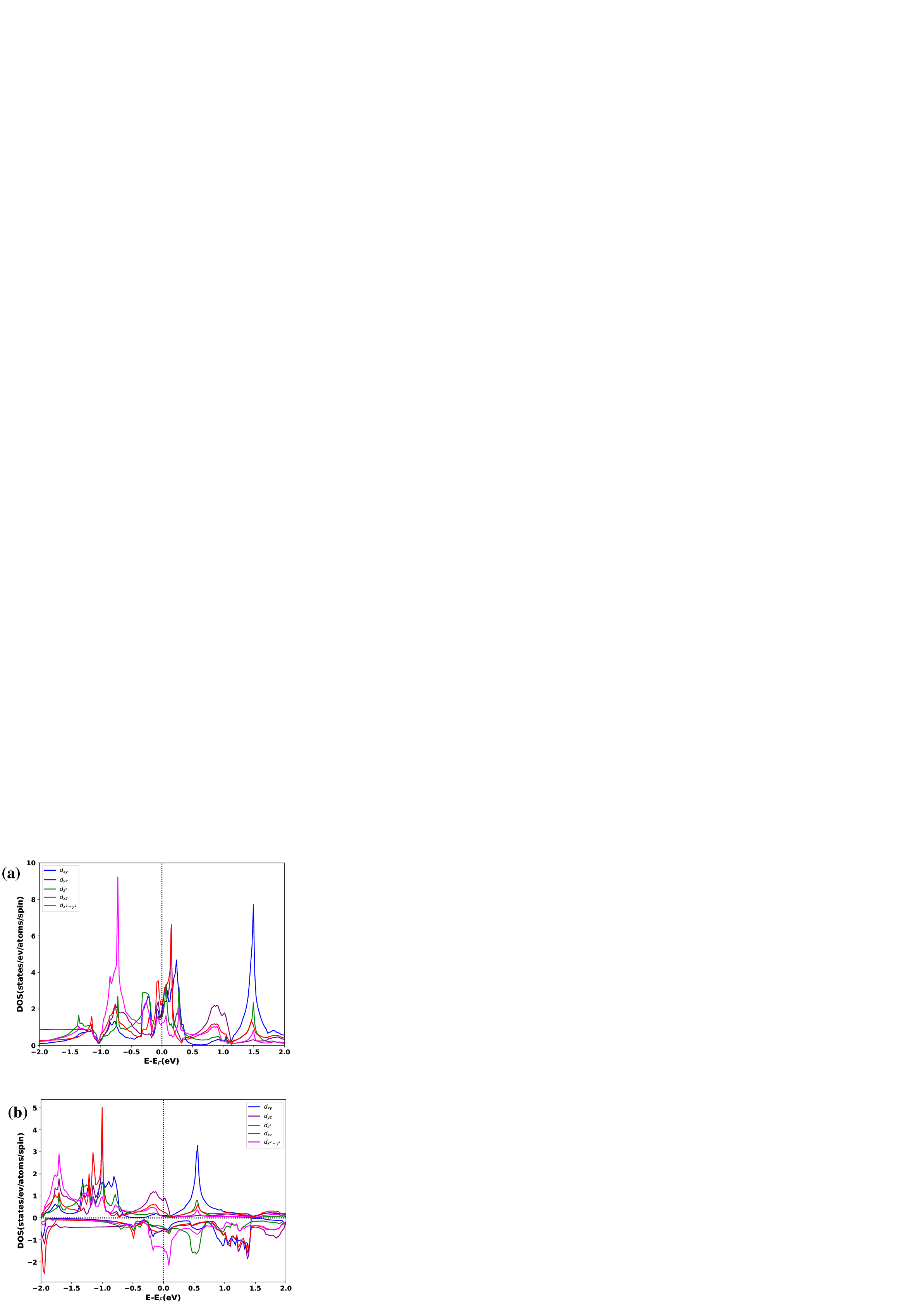


Figure S7: (a) DOS for (a) non spin polarized and (b) FM configuration calculated with GGA, where Mn_d_ states are shown with different colours.


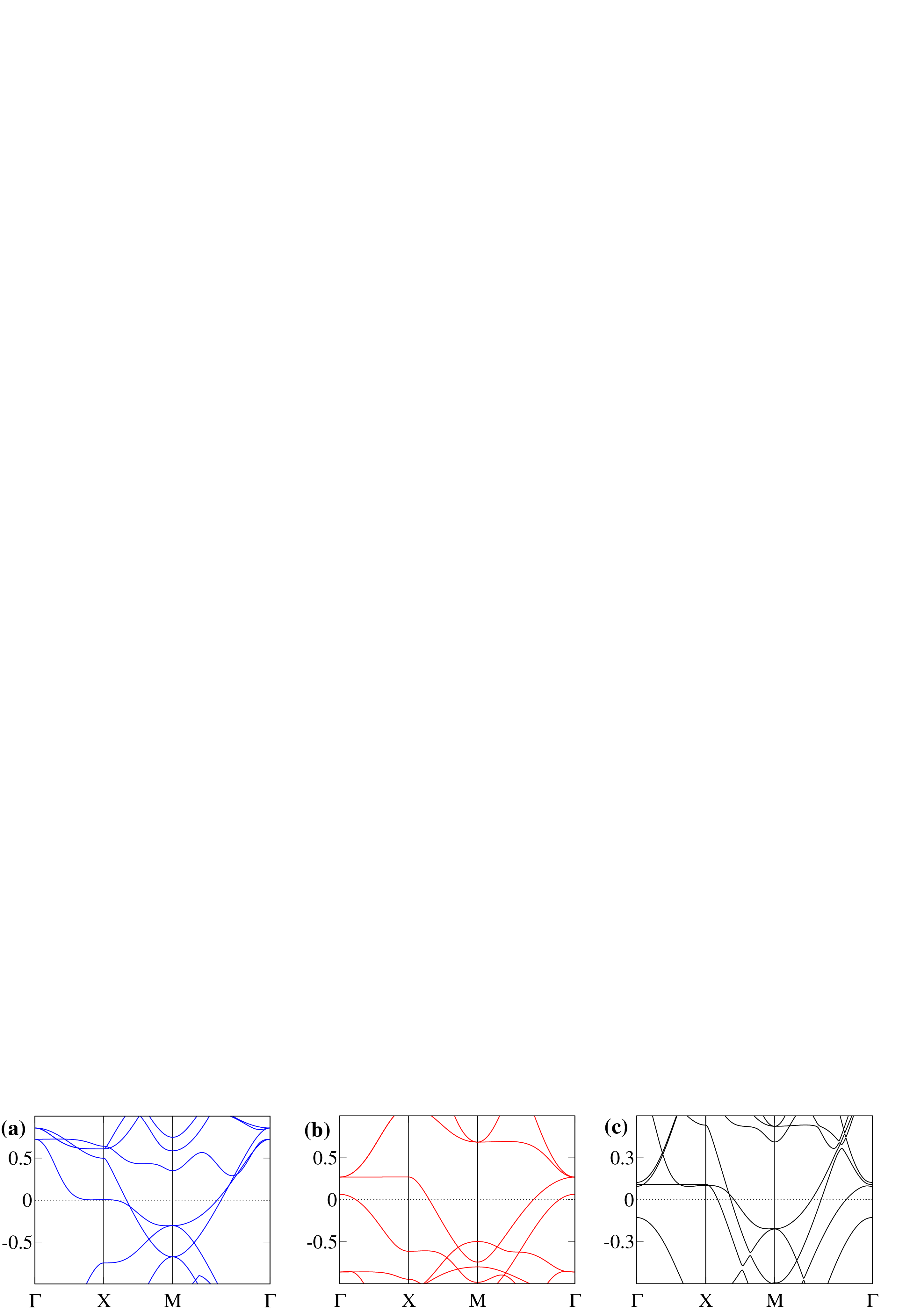


Figure S8: Band structure for FM configuration without SOC (a) spin-down, (b) spin-up and (c) with SOC having Mn moments along [001] in *k_x_* = 0 plane.

**References:**

1. Nakatsuji, S. *et al*. Large anomalous Hall effect in a non-collinear antiferromagnet at room temperature. Nature **527**, 212 (2015).
2. Heusler compounds Co2FeSi and Co2FeAl. *J. Appl. Phys.* **111**, 07D313 (2012).
3. Manyala, N. *et al.* Large anomalous Hall effect in a silicon-based magnetic semiconductor. *Nat. Mater.* **3**, 255 (2004).
4. Fang, Z. *et al*. The anomalous Hall effect and magnetic monopoles in momentum space. Science **302**, 92-95 (2003).
5. Kim, T. W., Lim, S. H. and Gambino, R. J. Spontaneous Hall effect in amorphous Tb–Fe and Sm–Fe thin films. *J. Appl. Phys.* **89**, 7212-7214 (2001).
6. Dijkstra, J. *et al*. Band-structure calculations of Fe_1/3_TaS_2_ and Mn_1/3_TaS_2_, and transport and magnetic properties of Fe_0.28_TaS_2_. J. Phys.: Condens. Matter **1**, 6363 (1989).
7. Nayak, A. K. *et al*. Large anomalous Hall effect driven by a nonvanishing Berry curvature in the noncolinear antiferromagnet Mn3Ge. Sci. Adv. **2**, e1501870 (2016).
8. Miyasato, T. *et al.* Crossover behavior of the anomalous Hall effect and anomalous Nernst effect in itinerant ferromagnets. *Phys. Rev. Lett.* **99**, 086602 (2007).
9. Kim, T. W. and Gambino, R. J. Composition dependence of the Hall effect in amorphous TbxCo1-x thin films. J. Appl. Phys. **87**, 1869-1873 (2000).
10. Ye, L. et al. Massive Dirac fermions in a ferromagnetic Kagome metal. Nature **555**, 638 (2018).
11. Yu, J. et al. Magnetotransport and magnetic properties of molecular-beam epitaxy L10 FePt thin films. J. Appl. Phys. **87**, 6854-6856 (2000).
